# Experimental and Theoretical Investigation of Hydrogen-Bonding Interactions in Cocrystals of Sulfaguanidine

**DOI:** 10.1021/acs.cgd.2c01337

**Published:** 2023-03-01

**Authors:** Shan Huang, Vinay K. R. Cheemarla, Davide Tiana, Simon E. Lawrence

**Affiliations:** † School of Chemistry, Synthesis and Solid State Pharmaceutical Centre, University College Cork, Cork T12 K8AF, Ireland; ‡ Analytical and Biological Chemistry Research Facility, University College Cork, Cork T12 K8AF, Ireland

## Abstract

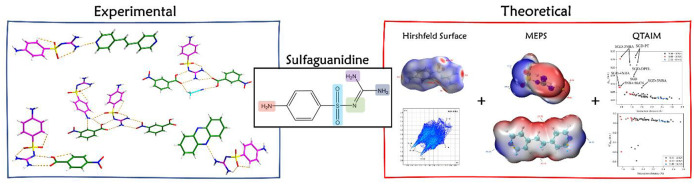

Pharmaceutical cocrystals, a type of multicomponent crystalline material incorporating two or more molecular and/or ionic compounds connected by noncovalent interactions (such as hydrogen bonds, π–π interactions, and halogen bonds), are attracting increasing attention in crystal engineering. Sulfaguanidine (SGD), one of the most frequently used sulfonamide compounds, was chosen as a model compound in this work to further investigate the hydrogen bond interactions in cocrystals, since it possesses various hydrogen bond donor and acceptor sites. Five cocrystals of SGD, synthesized successfully by slurry and slow evaporation methods, were fully characterized by thermal analysis, X-ray techniques, and Fourier transform infrared spectroscopy. To gain insight into the nature of hydrogen-bonding interactions, theoretical calculations including the analysis of Hirshfeld surface, MEPS (molecular electrostatic potential surface), and QTAIM (quantum theory of atoms in molecules) were conducted. The results are a part of a systematic study of cocrystals of sulfonamides that aims to establish synthon hierarchies in cocrystals containing molecules with multiple hydrogen-bonding functional groups.

## Abstract 2

Five cocrystals of sulfaguanidine were synthesized and fully characterized. Structural features and synthon hierarchy in the present cocrystals and three reported cocrystals are discussed. To gain insight into the nature of noncovalent interactions, theoretical calculations including the analysis of Hirshfeld surface, MEPS, and QTAIM were conducted.

## Introduction

Crystal engineering, as an important part of supramolecular chemistry, deals with the understanding of intermolecular interactions in the context of crystal packing and the utilization of such understanding in the design of new solids with expected physiochemical properties,^1^ which has been widely used in academia and industry, including pharmaceuticals,^2^ chemicals,^3^ photographic processing,^4^ textiles,^5^ electronics,^6^ etc. With the exploitation of crystal engineering strategies, recent decades have witnessed an enormous interest in the design of multicomponent crystalline materials (e.g. cocrystals, salts, hydrates/solvates).^7,8^ In particular, cocrystals offer many possibilities when it comes to crystal engineering since there are various coformers which can be assembled with target compounds by noncovalent interactions, such as hydrogen-bonding interactions, π–π interactions, halogen bonds, etc.^9^ These interactions can manipulate the molecular arrangement in crystal structures, resulting in the modification of the physicochemical properties, such as solubility, luminescence, stability, etc.^10−13^

Among different noncovalent intermolecular contacts, hydrogen bonds are of particular interest. Conventional hydrogen bonds (A–H···B, where A and B can be elements such as N, O, or F) represent the strongest interactions,^14^ while C–H···O/N belong to nonconventional hydrogen bonds, which are much weaker than conventional hydrogen bonds.^15^ Furthermore, the contribution of hydrogen bonds to the crystal stability is not an additive one; hence, one strong hydrogen bond interaction is not equivalent to the sum of several weak ones.^16^ In the late 1980s, Etter provided three general rules for hydrogen bond patterns in molecular solids,^17^ which are nowadays widely applied and proven to be of great use in the design and development of cocrystals. From the perspective of supramolecular chemistry, cocrystals can be considered as a structure composed of subunits (i.e., supramolecular synthons), the majority of which are joined by hydrogen bond interactions. Therefore, an in-depth understanding of hydrogen bond interactions will aid in the design of cocrystals with desired physicochemical properties.

More recently, several computational techniques have been developed to explain the intermolecular interactions in crystalline solids. The Hirshfeld surface is a unique tool to investigate and visualize different types of intermolecular interactions in crystals. The corresponding two-dimensional fingerprint plots can provide quantitative information on these interactions, for example, demonstrating common features and trends in specific compounds present in cocrystals.^18−20^ Luo et al. performed Hirshfeld surface analysis of pyrazinecarboxamide in 12 cocrystals and compared the quantitative information on intermolecular interactions with their crystal structures, revealing the influence in different contacts by different coformers.^19^ In addition, DFT calculations are also widely applied to elucidate whether cocrystal formation is possible in terms of the structure and interaction of molecules, gaining additional insight into complex information about intermolecular interactions in different cocrystals. As the formation of hydrogen bonds is primarily driven by electrostatic interactions, molecular electrostatic potential surfaces (MEPSs) can illustrate electrostatic interactions by visualizing the potential hydrogen donor and acceptor sites.^21,22^ Sarkar et al. synthesized eight cocrystals of thiophene-based compounds and conducted MEPS calculations, indicating that the prediction of the homomeric and heteromeric synthons matched the experimental cocrystallization studies in seven out of eight cases.^23^ In addition, QTAIM (quantum theory of atoms in molecules) analysis has been applied in the decoding of weak interactions in cocrystals, providing a pathway for comparing the experimental with the theoretically derived electron density based on the topological properties of the electron density (ρ).^24^ Bankiewicz and Wojtulewski investigated the molecular arrangement of dipicolinic acid with two coformers in their crystal structures by DFT and subsequent QTAIM analysis and obtained more detailed information about the topology and energy of interaction in the two cocrystals.^25^ Prediction of the stability based on the strongest intermolecular hydrogen bonds was performed on ten polymorphic drug systems using this methodology. It was found that predictions made with QTAIM analysis are more reliable than the ones made using the COMPASS force-field and DFT and DFT-D calculations.^16^

**Figure 1:**
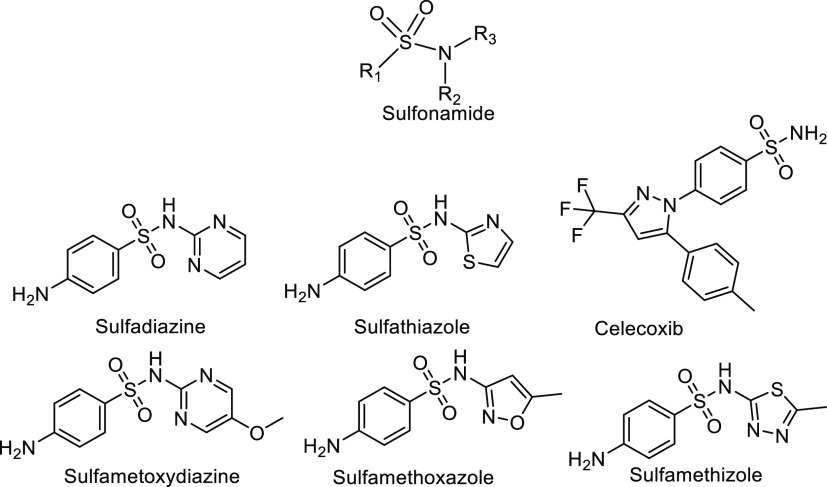
Chemical structures of some common sulfonamide drugs.

Sulfonamides are an important class of antibiotics used in veterinary and human medicine (Figure 1),^26^ and the crystal structure landscape of many sulfonamide drugs has been explored to date.^27^ For instance, Nangia et al. synthesized a series of cocrystals of celecoxib with carboxamide and investigated the synthons in different crystals and their physicochemical properties.^28^ MacGillivray and co-workers explored the cocrystals and salts of sulfadiazine and pyridines, demonstrating the “chameleon-like” behavior of tautomers at the cocrystal–salt boundary.^29^

**Figure 2:**
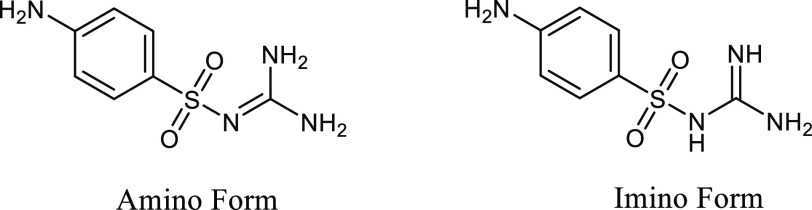
Molecular structures of sulfaguanidine (amino form on the left and imino form on the right).

Sulfaguanidine (SGD, Figure 2), one of the most frequently used sulfonamide compounds in medicated feeds, is used to treat enteric infections, such as bacillary dysentery.^30−32^ The amino group, sulfonyl group, and guanidyl group of the SGD molecule can participate in hydrogen bond interactions as donor and/or acceptor sites and form a variety of supramolecular synthons, which creates challenges in predicting supramolecular synthon behavior in different SGD crystal forms. To the best of our knowledge, the crystal form landscape of SGD has not been well explored yet. According to the Cambridge Structural Database (CSD) search,^33^ the first SGD-related structure SGD·H_2_O (CCDC refcode: 1261309) was published by Alléaume et al. in 1976, and demonstrated that the amino form of SGD is present in SGD·H_2_O while the imino form of SGD appears in some metal complexes of SGD (Figure 2).^34^ In 1977, three polymorphs of SGD (CCDC refcodes: 1317914–1317916) and SGD acetone solvate (CCDC refcode: 1317913) were published by Alberola et al.;^35^ however, their 3D structures are not available in the CSD and the original literature. The crystal structure of SGD (CCDC refcode: 1317917) was reported by Kálmán and co-workers in 1981.^36^ In 1986, the first SGD cocrystal SGD-ATP (antipyrine) (CCDC refcode: 1317714) was reported.^37^ Abidi et al. structurally analyzed two other SGD cocrystals: SGD-PT (1,10-phenantholine) and a dihydrate of SGD-TBA (thiobarbituric acid)·2H_2_O were analyzed structurally by Abidi and colleagues.^30^ These possess higher antibacterial activity and lower hemolytic toxicity compared with those of the starting materials.

**Figure 3:**
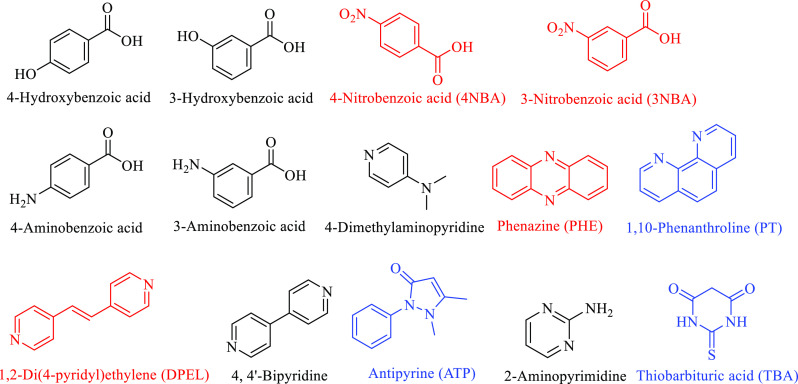
Molecular structures of coformers present in this work (successful are red, unsuccessful are black) and reported in the literature (blue).

In this study, five novel cocrystals of SGD with 1,2-di(4-pyridyl)ethylene (DPEL), 4-nitrobenzoic acid (4NBA), 3-nitrobenzoic acid (3NBA), and phenazine (PHE) (Figure 3) are reported. All of these cocrystals were fully characterized by thermal analysis, X-ray techniques, and FT-IR spectroscopy. To establish hierarchies of supramolecular synthons and gain an insight into the intermolecular interactions of SGD cocrystals, Hirshfeld surfaces, MEPS and QTAIM analyses were conducted on the five new SGD cocrystals as well as the three previously reported SGD cocrystals.

## Experimental Section

### Materials

Sulfaguanidine monohydrate was purchased from Fluorochem and used as received without further purification. All of the coformers were purchased from Sigma-Aldrich and used as received. Solvents were obtained from commercial sources and used as received without further purification.

### Synthesis

#### SGD-DPEL

For the crystallization experiment, a 1:1 molar ratio of SGD·H_2_O (35.5 mg, 0.15 mmol) and DPEL (27.9 mg, 0.15 mmol) was placed in a mixture of acetonitrile and methanol (1:1, v/v). Colorless platelike crystals were harvested after 3–5 days. A 1:1 mixture of SGD·H_2_O (231.9 mg, 1 mmol) and DPEL (182.5 mg, 1 mmol) in methanol was used for the slurry experiments.

#### SGD-4NBA

Crystals were produced by dissolving a 1:1 molar ratio of SGD·H_2_O (35.2 mg, 0.15 mmol) and 4NBA (25.5 mg, 0.15 mmol) in methanol to yield yellow platelike crystals after 3–5 days. A 1:1 mixture of SGD·H_2_O (232.4 mg, 1 mmol) and 4NBA (166.5 mg, 1 mmol) in methanol was used for the slurry experiments.

#### SGD-3NBA

Crystals were produced by dissolving a 1:1 molar ratio of SGD·H_2_O (34.9 mg, 0.15 mmol) and 3NBA (25.2 mg, 0.15 mmol) in methanol to afford yellow platelike crystals after 3–5 days. For the slurry experiments, a 1:1 mixture of SGD·H_2_O (232.1 mg, 1 mmol) and 3NBA (167.7 mg, 1 mmol) in methanol was used.

#### SGD-3NBA·MeCN

The cocrystal solvate was produced by dissolving a 1:2 molar ratio of SGD·H_2_O (35.2 mg, 0.15 mmol) and 3NBA (50.8 mg, 0.3 mmol) in a mixture of acetonitrile and deionized water (1:1, v/v). Yellow platelike crystals were harvested after 3–5 days. Bulk amounts of the solvated cocrystal were made by slurrying a 1:2 mixture of SGD·H_2_O (232.5 mg, 1 mmol) and 3NBA (335.1 mg, 2 mmol) in acetonitrile for 3 days.

#### SGD-PHE

Yellow platelike crystals were produced by dissolving a 1:1 molar ratio of SGD·H_2_O (35.1 mg, 0.15 mmol) and PHE (26.8 mg, 0.15 mmol) in ethanol and harvesting after 1–2 days. A 1:1 mixture of SGD·H_2_O (229.8 mg, 1 mmol) and PHE (179.8 mg, 1 mmol) in ethanol was used for the slurrying experiments.

### Physical Measurements

Differential scanning calorimetry (DSC) experiments were conducted on a TA Instruments Q1000 instrument under a continuously purged dry nitrogen atmosphere. Powdered samples (2–6 mg) were crimped in nonhermetic aluminum pans and analyzed from 25 to 300 °C at a heating rate of 10 °C min^–1^. IR spectra were obtained on a PerkinElmer UATR Two spectrophotometer using a diamond attenuated total reflectance accessory. Powdered samples were scanned over a range of 400–4000 cm^–1^, and an average of four scans were taken for each spectrum obtained with a resolution of 4 cm^–1^. PXRD data were recorded by using a STOE STADI MP diffractometer with Cu Kα radiation using a linear position-sensitive detector over the 2θ range of 3.5–45.5° with an increment of 0.05° at a rate of 2° min^–1^. The powdered samples were made between transmission foils, and the data were analyzed via STOE WinXPOW POWDAT software.^38^ Single crystal X-ray diffraction (SCXRD) data were collected on a Bruker APEX II DUO instrument with monochromated Mo Kα radiation (λ = 0.7107 Å). All calculations and refinements were made using Bruker APEX software with the SHELX suite of programs.^39,40^ Nonhydrogen atoms were refined anisotropically. All hydrogen atoms were placed in geometrically calculated positions using the riding model, with C–H = 0.93–0.97 Å and N–H = 0.86–0.89 Å and Uiso (H) (in the range 1.2–1.5 times Ueq of the parent atom). Crystal structures were viewed and analyzed using the DIAMOND 4.6 software package,^41^ and the data of potential hydrogen bonds and π–π interactions were obtained using the PLATON program.^42,43^ Crystallographic parameters are listed in Table 1.

**Table 1:** Crystallographic Data for SGD-DPEL, SGD-4NBA, SGD-3NBA, and SGD-3NBA·MeCN Cocrystals

	SGD-DPEL 1:1	SGD-4NBA 1:1	SGD-3NBA 1:1	SGD-3NBA·MeCN 1:2:1	SGD-PHE 1:1
chemical formula	C_19_H_20_N_6_O_2_S	C_14_H_15_N_5_O_6_S	C_14_H_15_N_5_O_6_S	C_23_H_23_N_7_O_10_S	C_19_H_18_N_6_O_2_S
formula weight	396.47	381.37	381.37	589.54	394.45
crystal system	triclinic	monoclinic	monoclinic	triclinic	monoclinic
space group, *Z*	*P*1̅, 2	*C*2/*c*, 8	*C*2/*c*, 8	*P*1̅, 2	*P*2_1_/*c*, 4
temperature (K)	296(2)	296	296	296(2)	296(2)
*a* (Å)	9.2182(6)	29.597(7)	23.0253(18)	8.3188(10)	8.7472(5)
*b* (Å)	10.1853(7)	7.0348(17)	12.2300(9)	10.4106(14)	13.0128(9)
*c* (Å)	11.3564(8)	16.074(4)	14.775(2)	16.261(2)	16.8853(9)
α (deg)	84.599(2)	90	90	105.555(3)	90
β (deg)	79.857(2)	96.582(5)	124.408(1)	90.390(4)	102.097(2)
γ (deg)	66.6940(10)	90	90	102.618(2)	90
volume (Å^3^)	963.60(11)	3324.7(14)	3432.7(6)	1320.7(3)	1879.3(2)
ρ_calc_ (g cm^–3^)	1.366	1.524	1.476	1.483	1.394
radiation type	Mo Kα	Mo Kα	Mo Kα	Mo Kα	Mo Kα
μ (mm^–1^)	0.196	0.240	0.232	0.193	0.201
reflns measured	18145	14795	16726	25438	18949
reflns independent	4808	4171	4294	6638	4685
significant [*I* > 2σ(*I*)]	4150	3378	3647	4922	3651
parameters refined	277	256	256	373	253
restraints	18	13	13	24	0
Δρ_max_, Δρ_min_ (e Å^–3^)	0.25, −0.47	0.39, −0.64	0.26, −0.63	0.389, −0.393	0.357, −0.327
*F*(*000*)	416	1584	1584	612	824
*R*_1_ [*I* > 2σ(*I*)]	0.0379	0.0501	0.0364	0.0547	0.0424
w*R*_2_ (all data)	0.1076	0.1719	0.1070	0.1647	0.1063
CCDC number	2190690	2190688	2190691	2190689	2190692

### Computational Studies

Hirshfeld surface analysis and two-dimensional fingerprint plots were analyzed by using the CrystalExplorer 21.5 program.^20^ The optimized geometries and energies of the SGD cocrystals in the ground state were obtained by the density functional theory (DFT) methods using the Gaussian 09 program package employing the B3LYP functional with the 6-311G (d,p) basis set.^21^ MEPSs were computed at the same level of theory using the Multiwfn 3.8 program and plotted using VMD.^24,44,45^ QTAIM analysis was carried out on geometry optimized cocrystal structures using a periodic plane wave DFT using the QE package.^46^ Ultrasoft pseudopotential with kinetic energy cutoff 45 and 425 Ry charge density cutoff was employed for the calculations. Critic2 was used for topology analysis to locate the bond critical points.^47^ Estimated hydrogen bonding energy was calculated from the theoretical equation fitted with experimental data.^48^

## Results and Discussion

### Physical Characterization

The thermal behavior of the SGD cocrystals was assessed using DSC. The DSC traces of the five obtained cocrystals and the corresponding single components are shown in Figure S1. Table 2 displays the melting points of the five cocrystals and their starting materials. SGD-DPEL and SGD-PHE cocrystals melt at a higher temperature than the starting materials, while the melting points of SGD-4NBA, SGD-3NBA, and SGD-3NBA·MeCN are in between those of the individual components. IR spectra of the cocrystals and starting materials are shown in Figure S2−S5. As shown in Table 3, the —NH_2_, C=N, and sulfonyl group bands of SGD·H_2_O exhibit a blue shift in all five cocrystals. Meanwhile, all the observed differences indicate that the sulfonyl group, amino group, and/or guanidyl group are involved in the formation of hydrogen bonds in different cocrystals, confirming the formation of the new crystalline forms of SGD.

**Table 2:** Melting Points of Cocrystals and Starting Materials

solids	*T*_m_ (°C)	solids	*T*_m_ (°C)
SGD·H_2_O	189–190^49^	SGD-DPEL	193–196
DPEL	150–153^50^	SGD-4NBA	218–220
4NBA	240–241^51^	SGD-3NBA	178–181
3NBA	142–143^52^	SGD-3NBA·MeCN	175–178
PHE	175–176^53^	SGD-PHE	209–211

**Table 3:** Distinctive Bands (cm^–1^) in the FT-IR Spectra of SGD·H_2_O and the Cocrystals

solid form	νNH_2_	νSO_2_	ν_C=N_
SGD·H_2_O	3394, 3337	1123, 1082	1612
SGD-DPEL	3398, 3339	1132, 1082	1634
SGD-4NBA	3407, 3368	1130, 1090	1627
SGD-3NBA	3438, 3367	1136, 1094	1629
SGD-3NBA·MeCN	3454, 3374	1133, 1092	1625
SGD-PHE	3401, 3358	1122, 1088	1634

**Figure 4:**
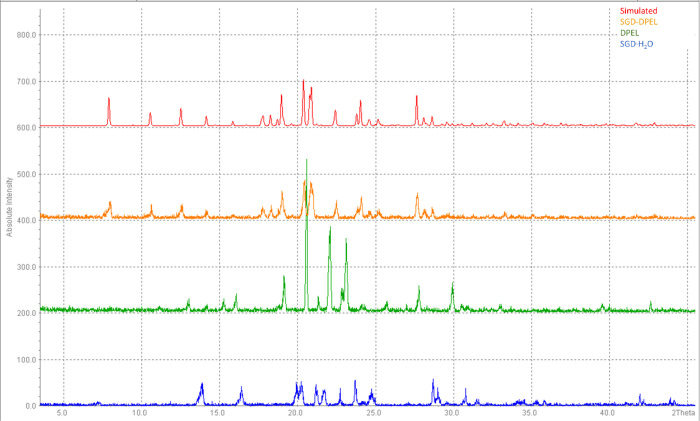
PXRD patterns of SGD·H_2_O (blue), DPEL (green), and SGD-DPEL (orange) and simulated pattern from the crystal structure (red).

PXRD patterns of SGD-DPEL, the two starting materials, and a simulated pattern from the SCXRD analysis are shown in Figure 4. SGD-DPEL cocrystal exhibits several new diffraction peaks at 2θ values of 7.9°, 10.5°, 12.7°, etc., which are not present in the patterns of the two starting materials, suggesting the formation of new crystalline forms. The PXRD patterns of the other four cocrystals are displayed in Figure S6. All of the PXRD patterns of the five cocrystals match with the simulated patterns extracted from the SCXRD analysis, indicating these cocrystals can be reproduced in bulk quantities by the slurry method.

### Crystal Structure Analysis

Single crystals of the five cocrystals were obtained, and their structures were determined by SCXRD. Ellipsoid plots are shown in Figure S7. Hydrogen bonds and π–π interaction geometries are displayed in Tables S1–S5, separately.

#### SGD-DPEL

**Figure 5:**
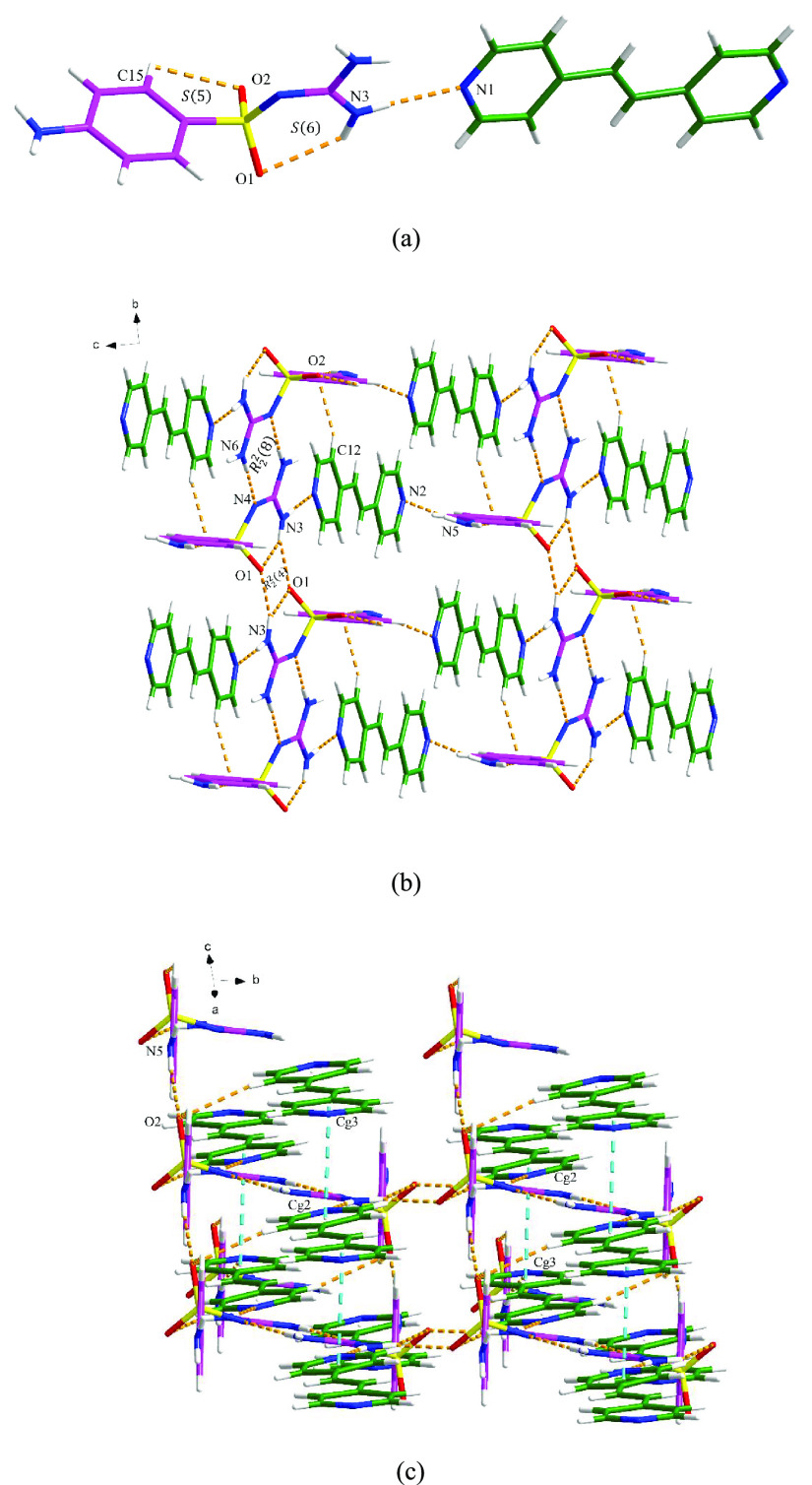
Crystal packing diagrams of the SGD-DPEL cocrystal: (a) asymmetric unit (pink is SGD and green is DPEL), (b) two-dimensional hydrogen-bonding network, and (c) three-dimensional network (hydrogen bonding is displayed by orange dashed lines, and π–π interaction is displayed by blue dashed lines).

The SGD-DPEL cocrystal crystallizes in the triclinic space group *P*1̅. The asymmetric unit consists of one SGD molecule and one DPEL molecule. As shown in Figure 5a, the two components interact with each other via a discrete N3–H11···N1 hydrogen bond. Along the *a* axis, an *R*_2_^2^(8) motif and an *R*_2_^2^(4) ring are generated by two SGD molecules through N6–H14···N4 and N3–H20···O1 hydrogen bond interactions. The 2D hydrogen-bonding network is extended via C–H···O and N–H···N discrete hydrogen bonds (Figure 5b). The 3D crystal lattice is stabilized by the N5–H15···O2 hydrogen bond interaction and the π–π interactions through the pyridyl rings of DPEL molecules between the layers (Figure 5c).

#### SGD-4NBA

**Figure 6:**
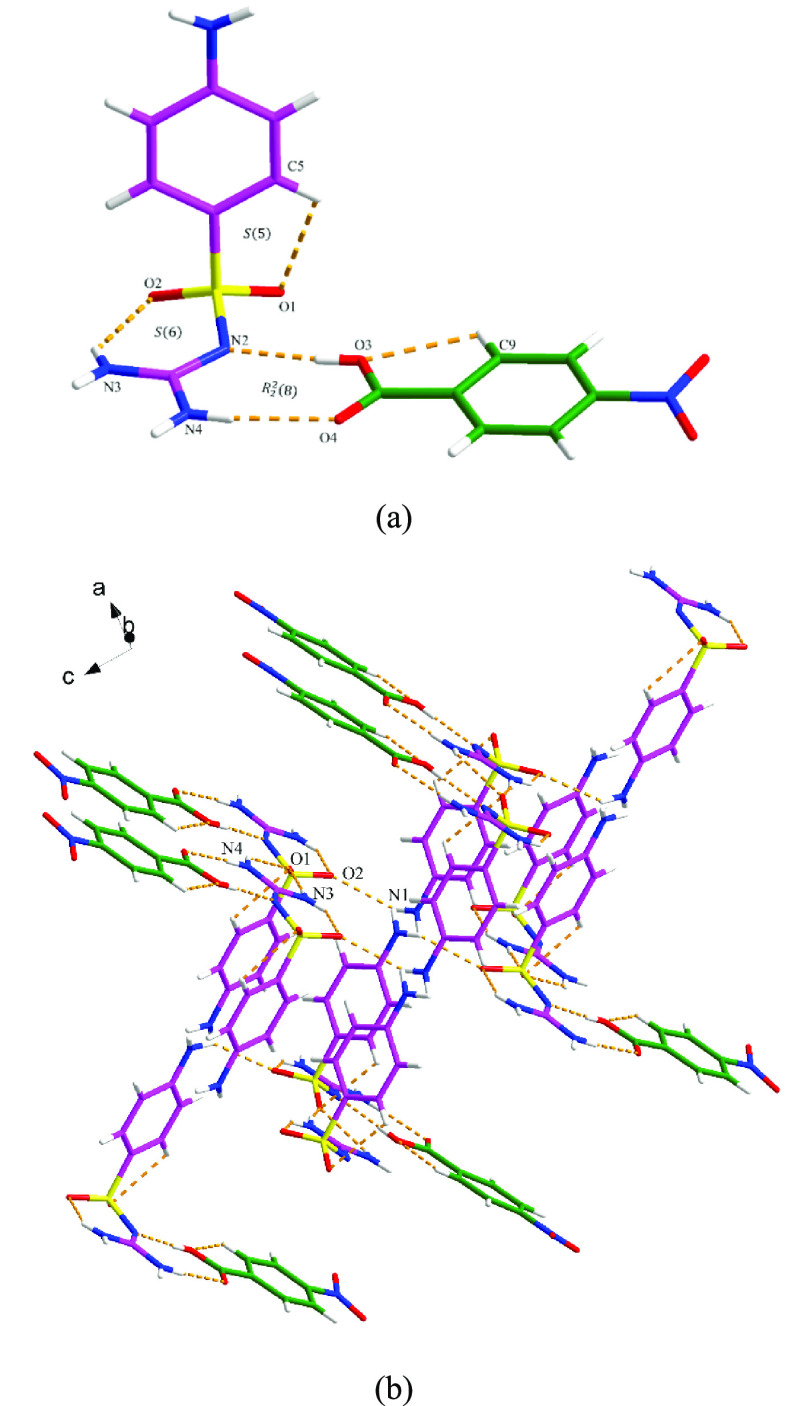
Crystal packing diagrams of the SGD-4NBA cocrystal: (a) asymmetric unit (pink is SGD and green is 4NBA) and (b) three-dimensional hydrogen-bonding network (hydrogen bonding is displayed by orange dashed lines).

SGD and 4NBA form a cocrystal that crystallizes with one SGD molecule and one 4NBA molecule in the asymmetric unit. As shown in Figure 6a, the two components interact with each other through discrete N4–H17···O4 and O3–H15···N2 hydrogen bonds, resulting in an *R*_2_^2^(8) motif. The basic unit is extended via three discrete N–H···O hydrogen-bonding interactions, i.e., N3–H13···O1, N4–H16···O1, and N1–H18···O2, resulting in the 3D hydrogen-bonding network (Figure 6b). No π–π interactions participate in stabilizing the three-dimensional structure of the SGD-4NBA cocrystal.

#### SGD-3NBA

**Figure 7:**
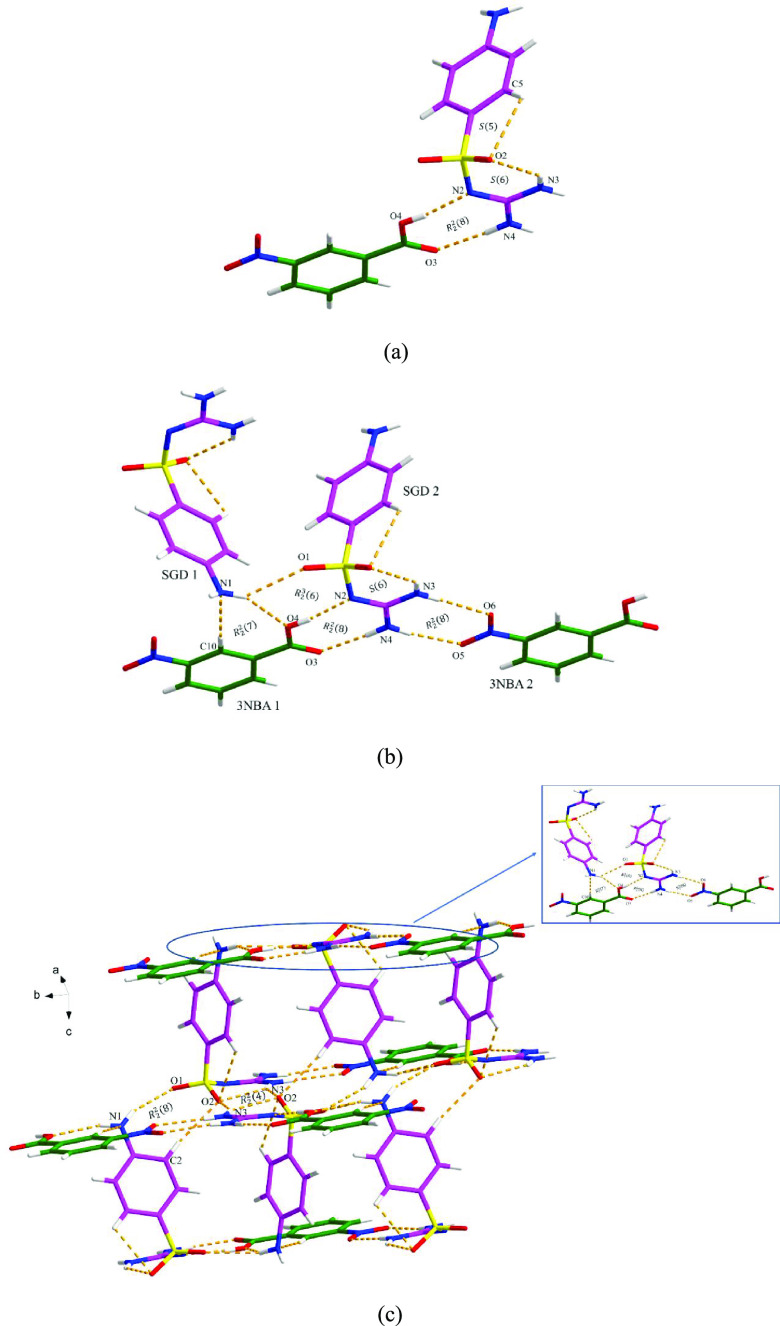
Crystal packing diagrams of the SGD-3NBA cocrystal: (a) asymmetric unit (pink is SGD and green is 3NBA), (b) four motifs between two SGD molecules and 3NBA molecules, and (c) three-dimensional hydrogen-bonding network (hydrogen bonding is displayed by orange dashed lines).

The SGD-3NBA cocrystal crystallizes in the monoclinic system with the *C*2/*c* space group. The asymmetric unit contains one SGD molecule and one 3NBA molecule (Figure 7a). As shown in Figure 7b, four continuous motifs are produced between two SGD molecules and two 3NBA molecules. Specifically, the amino group of SGD 1, the sulfonyl guanidyl group of SGD 2, the carboxyl group from 3NBA 1 and the nitro group from 3NBA 2 are involved in the formation of (from left to right) an *R*_2_^2^(7) motif, an *R*_2_^3^(6) motif, and two *R*_2_^2^(8) motifs via N–H···O, O–H···N, and C–H···N discrete hydrogen bond interactions. The 3D structure is further assembled by the N–H···O and C–H···O discrete hydrogen bond interactions (Figure 7c). No additional π–π interactions contribute to the extended 3D structure.

#### SGD-3NBA·MeCN

**Figure 8:**
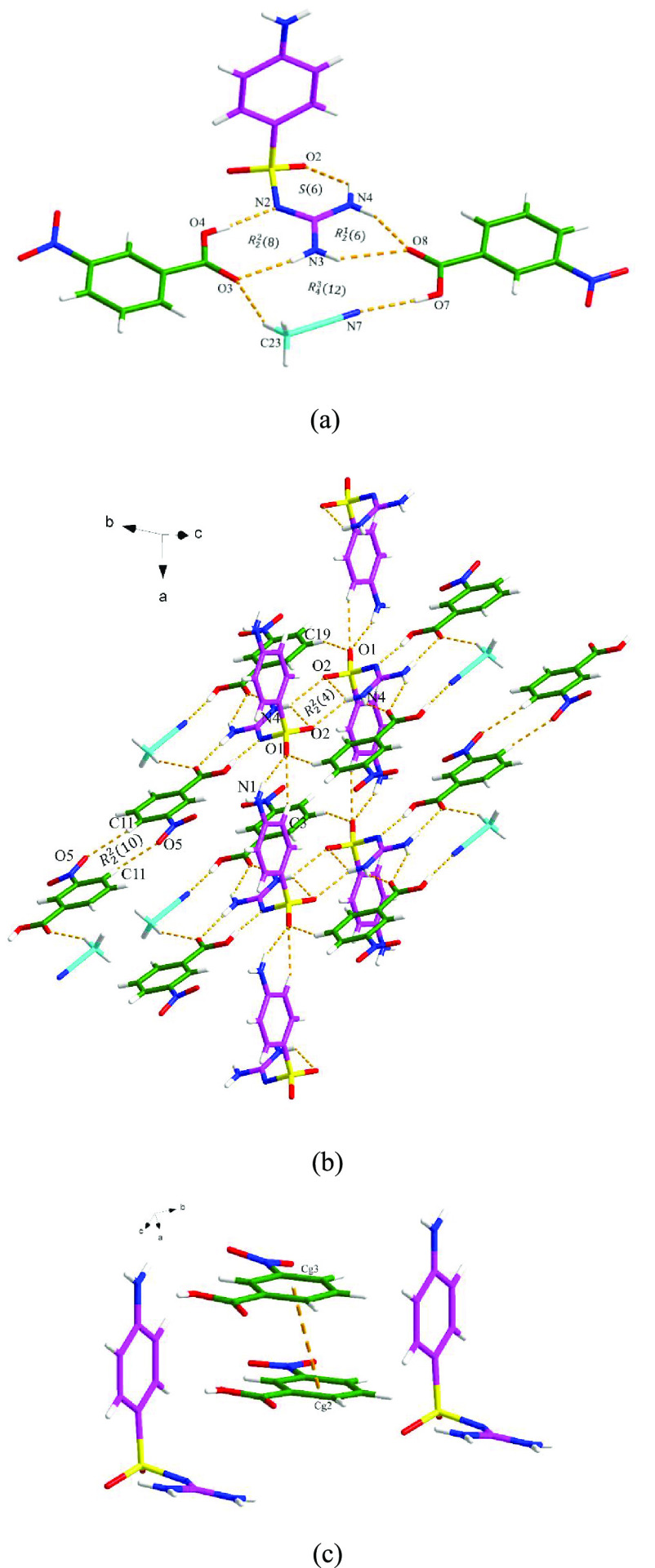
Crystal packing diagrams of the SGD-3NBA·MeCN cocrystal: (a) asymmetric unit (pink is SGD, green is 3NBA and blue is MeCN), (b) hydrogen-bonding network (hydrogen bonding is displayed by orange dashed lines), and (c) the three-dimensional network resulting from interstack π–π interactions as shown by orange dashed lines (hydrogen bonding is not displayed for clarity).

SGD-3NBA·MeCN crystallizes in the triclinic space group *P*1̅ with one molecule of SGD, two molecules of 3NBA, and one molecule of MeCN in the asymmetric unit. As shown in Figure 8a, *R*_2_^2^(8), *R*_2_^1^(6), and *R*_4_^3^(12) motifs are generated among these four components via N–H···O, C–H···O, and O–H···N discrete hydrogen bond interactions. The hydrogen-bonding network is extended via the C–H···O and N–H···O hydrogen bond interactions, with the latter hydrogen bond interactions forming an *R*_2_^2^(4) ring. Furthermore, two 3NBA molecules are linked by two C11–H11···O5 discrete hydrogen bond interactions, resulting in an *R*_2_^2^(10) motif (Figure 8b). The 3D structure is also strengthened by π–π interactions through the phenyl rings of the 3NBA molecules between the layers (Figure 8c).

#### SGD-PHE

**Figure 9:**
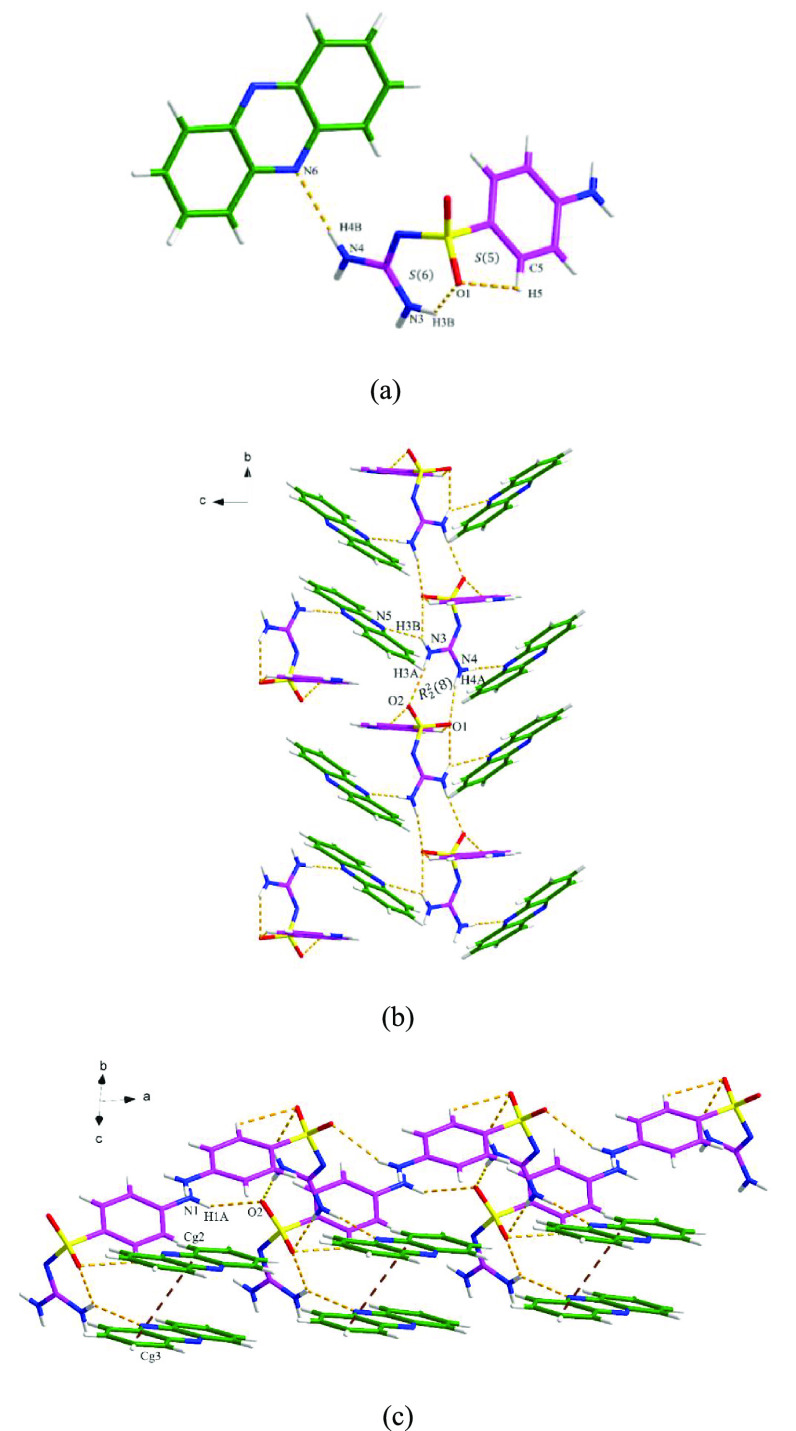
Crystal packing diagrams of the SGD-PHE cocrystal: (a) asymmetric unit (pink is SGD and green is PHE), (b) hydrogen-bonding network (hydrogen bonding is displayed by orange dashed lines), and (c) three-dimensional network resulting from interstack π–π interactions, as shown by red-brown dashed lines.

SGD-PHE cocrystallizes in the *P*2_1_/*c* space group with *Z* = 4, the asymmetric unit consisting of one SGD molecule and one PHE molecule (Figure 9a). Notably, the guanidyl group of the SGD molecule is only involved as a hydrogen donor in this cocrystal. Along the *a* axis, the guanidyl group of SGD links the PHE molecule via two N–H···N discrete hydrogen bond interactions. The 3D structure is extended through N3–H3A···O2 and N4–H4A···O1 hydrogen-bonding interactions, forming an *R*_2_^2^(8) motif (Figure 9b). The N1–H1A···O2 hydrogen-bonding interactions between the amino group and sulfonyl group of the SGD molecule and the additional π–π interactions between the phenyl rings and pyrazine rings from PHE also contribute to the extended 3D structure of the SGD-PHE cocrystal (Figure 9c).

The structural analysis of SGD-ATP^37^ was also conducted prior to the investigation of the hydrogen-bonding interactions of SGD cocrystals. Hydrogen bond and π–π interaction geometries are displayed in Table S6.

#### SGD-ATP

**Figure 10:**
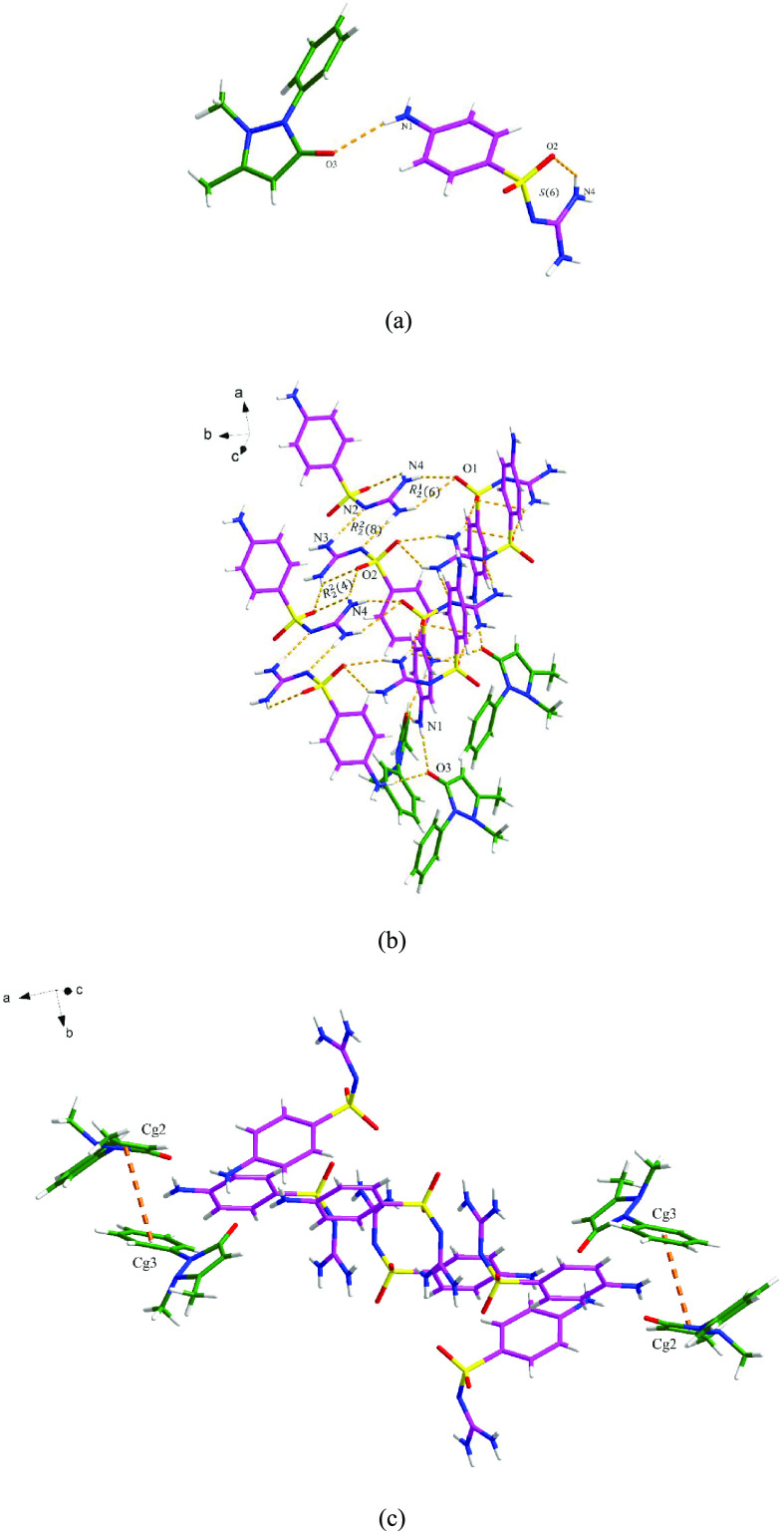
Crystal packing diagrams of the SGD-ATP cocrystal: (a) asymmetric unit (pink is SGD and green is ATP), (b) hydrogen-bonding network (hydrogen bonding is displayed by orange dashed lines), and (c) three-dimensional network resulting from interstack π–π interactions, as shown by orange dashed lines (hydrogen bonding is not displayed for clarity).

SGD-ATP cocrystallizes in the monoclinic crystal system, the *P*2_1_/*c* space group, with one SGD molecule and ATP molecule in the asymmetric unit, which interact via the N1–H3···O3 hydrogen bond interaction (Figure 10a). As shown in Figure 10b, the same ATP molecule connects with another adjacent SGD molecule through the N1–H3···O3 hydrogen bond interaction. The crystal structure is further extended by the hydrogen bond interactions between SGD molecules, generating three different motifs. Specifically, an *R*_2_^1^(6) motif is formed via N3–H7···O1 and N4–H8···O1, and an *R*_2_^2^(4) motif is formed via N4–H9···O2 inter- and intrahydrogen bond interactions between the guanidyl and sulfonyl groups in SGD. In addition, an *R*_2_^2^(8) homosynthon is generated through N3–H6···N2 from the two guanidyl groups. The 3D structure is also stabilized by π–π interactions through the pyrazole and phenyl rings of ATP molecules between the layers (Figure 10c).

**Figure 11:**
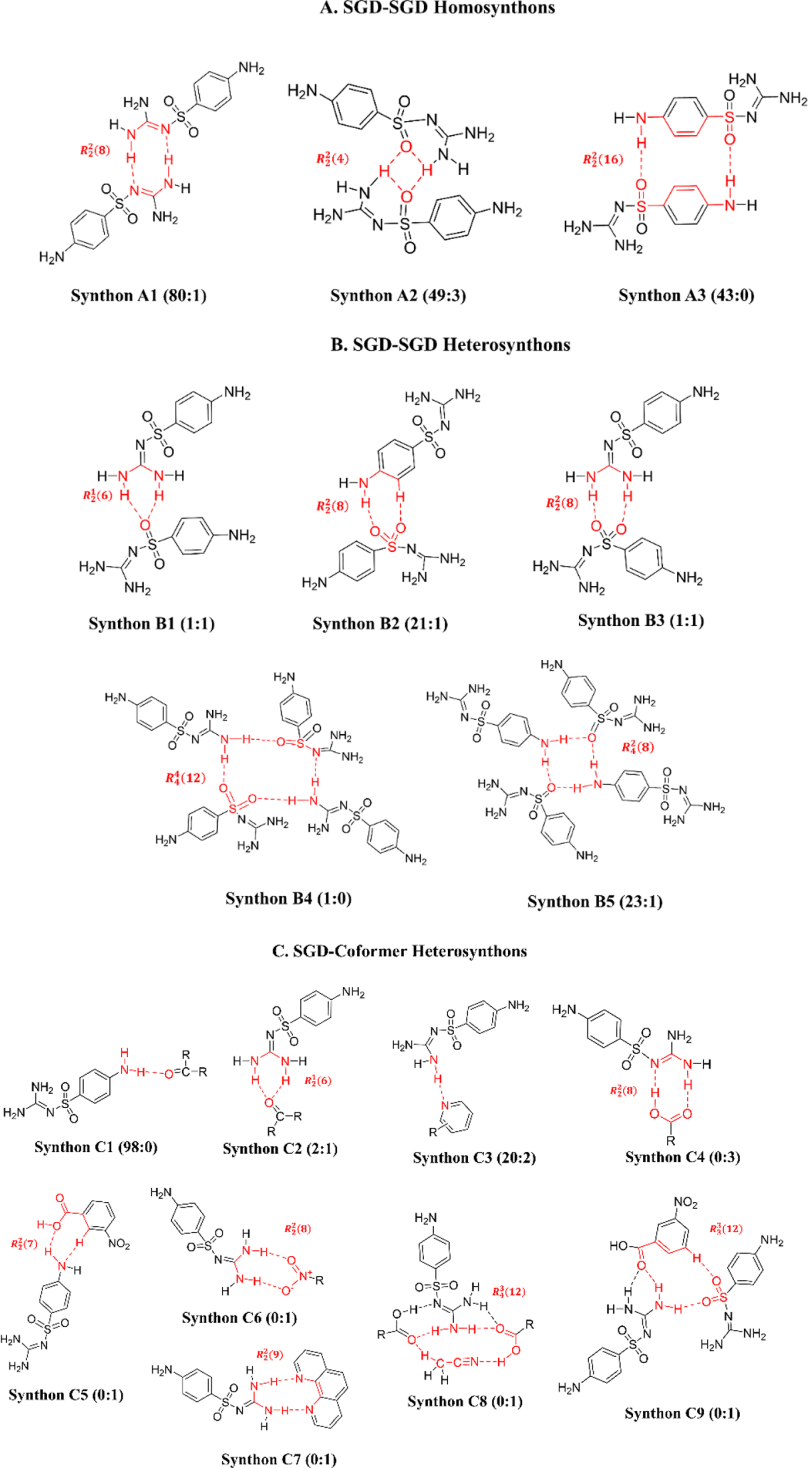
Types of synthons identified in the eight SGD cocrystals. Numbers indicate the number of occurrences in the CSD (left) and in this work (right).

Based on the structural analysis above, the SGD molecule can form different types of synthons with either SGD molecules or coformer molecules due to the various kinds of hydrogen bond donor and acceptor sites in the molecules. Figure 11 illustrates the different types of supramolecular synthons^54^ in the SGD cocrystals, their graph set notation,^55,56^ and the frequency of occurrence of each synthon in multicomponent solids of sulfonamides deposited in CSD. The results of the CSD search were obtained and filtered by 3D coordinates determined, only single crystal structures, and only organics using ConQuest (version 2022.2.0). Table 4 displays the frequency of occurrence of each synthon in the different SGD cocrystals. Among all SGD-SGD synthons, synthon A1 has been widely reported in the multicomponent solids of sulfonamides in the CSD. However, this robust synthon is less likely to occur when forming cocrystals with acids since in these cases the SGD–coformer interactions might be relatively weaker, resulting in the failure of cocrystal formation.^57^ In this work, synthon A1 was observed in SGD-DPEL and SGD-PT cocrystals, which is reasonable as these coformers can form strong interactions with SGD through the extra hydrogen atoms from the guanidyl group, resulting in the formation of robust synthon C3. When cocrystallizing with benzoic acid or its derivatives, the guanidyl group from SGD is more likely to form a heterosynthon (C4) with the carboxylic group from the acid instead of forming a homosynthon (A1) with the guanidyl group from another SGD, which is usually energetically more favored.^58,59^ Synthons C5 to C9 are specific to the structures of the coformers discussed in this work.

**Table 4:** List of the Occurrence of Synthons in SGD Cocrystals

	synthon	SGD-DPEL^a^	SGD-4NBA^a^	SGD-3NBA^a^	SGD-3NBA·MeCN^a^	SGD-PHE^a^	SGD-PT^30^	SGD-TBA·2H_2_O^30^	SGD-ATP^37^
SGD-SGD	A1	√					√		√
	A2	√		√	√				√
	A3							√	
	B1		√						√
	B2			√					
	B3					√			
	B4								√
	B5			√					
SGD–coformer	C1								√
	C2				√			√	
	C3	√				√	√		
	C4		√	√	√				
	C5			√					
	C6			√					
	C7						√		
	C8				√				
	C9				√				

a Crystal structure obtained in this work.

### Computational Studies

#### Hirshfeld Surface Analysis

**Figure 12:**
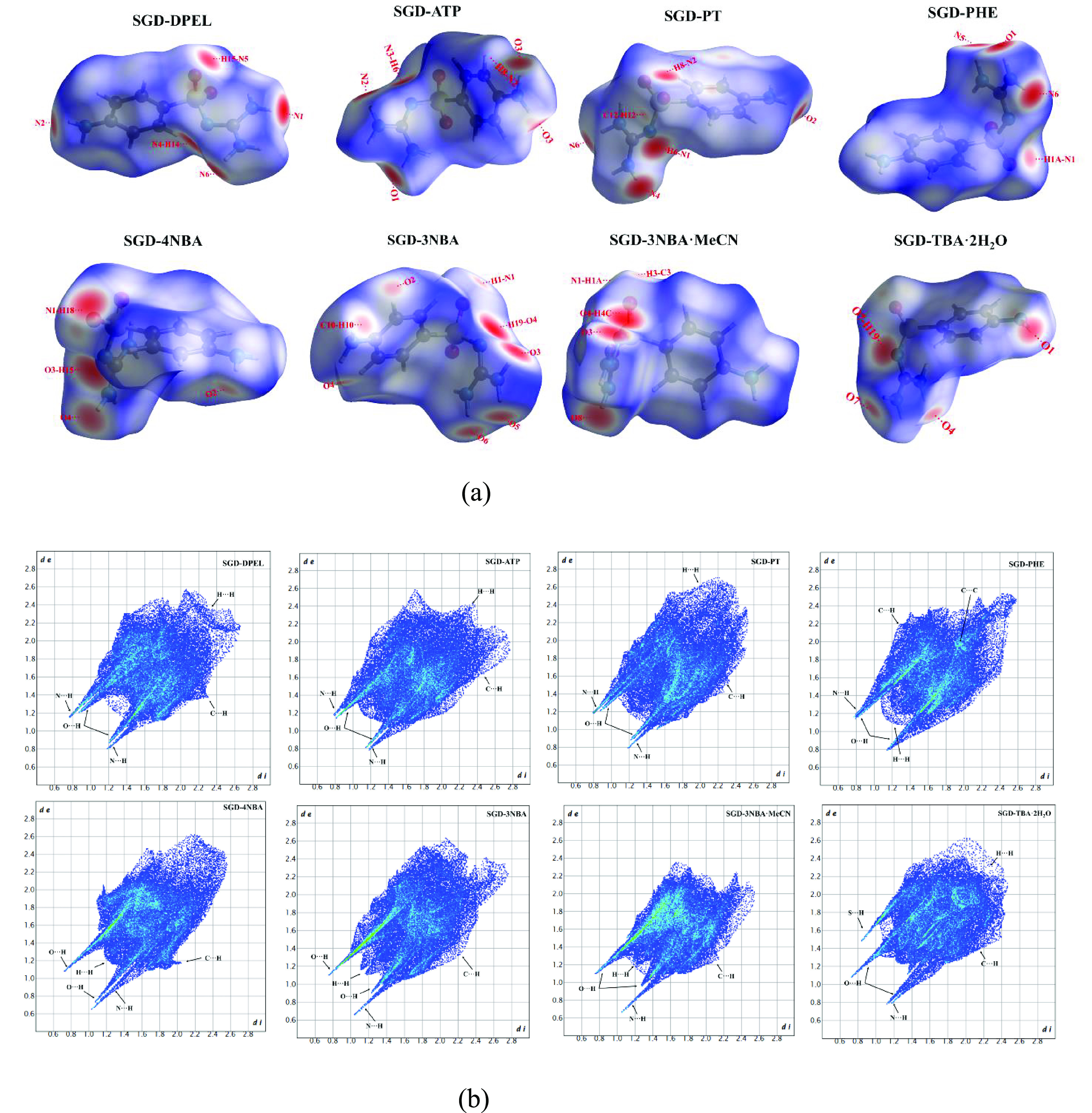
(a) Hirshfeld surfaces and (b) 2D fingerprint plots of SGD in SGD cocrystals.

The Hirshfeld surface analysis has been utilized to investigate and visualize different types of intermolecular interactions in the crystal, and the 2D fingerprint plots provide quantitative information about these interactions.^18,19^Figure 12a illustrates the Hirshfeld surfaces of SGD that have been mapped over *d*_norm_, where the large circular depressions (deep red) stand for the hydrogen bonding contacts (i.e., H···O and H···N) whereas other visible spots represent the H···H contacts.^60^Figure 12b demonstrates the corresponding 2D fingerprint plots. In particular, the hydrogen bonding contacts (appearing as spikelike tips), H···H contacts (appearing as asymmetric points spread over a large area), and the C···H contacts (presented as a symmetric pair of wings) are the three most significant contacts in the eight cocrystals.^19^

According to the structural analysis and Table 4, the SGD molecule in any SGD cocrystals can interact with not only coformer molecules but also other SGD molecules. However, the majority of coformer molecules only interact with SGD molecules in the SGD cocrystals. The exceptions involve three component systems, namely, SGD-3NBA·MeCN, this work, and SGD-TBA·2H_2_O.^30^ Therefore, the various contact contributions of coformers were obtained to investigate the influence of different coformers on the intermolecular interactions of SGD molecules in different SGD cocrystals (Table 5).

**Table 5:** Summary of the Various Contact Contributions in SGD Cocrystals (%)

		hydrogen bond						
	H···H	H···N	H···O	C···H	C···C	N···C	O···C	others
SGD-ATP	50.6	7.5	15.4	22.1	3.9	0.1	0.2	0.2
SGD-PHE	46.9	12.7	4.8	22.4	8.3	4.3	0.2	0.4
SGD-DPEL	44.5	13.1	5.4	27.1	2.5	6.4	0.1	0.9
SGD-PT	39.9	16	4.9	24.9	9.1	4.5	0.6	0.1
SGD-4NBA	19.2	7.5	41	12.6	4.2	0.4	12.1	3
SGD-3NBA	15.3	6.6	33.2	25.4	0.1	2	10.2	7.2

As shown in Table 5, H···H, hydrogen-bonding interactions (N···H and O···H), and C···H are the three significant contacts. For SGD-ATP, SGD-PHE, SGD-DPEL, and SGD-PT cocrystals, H···H contacts make the largest contribution, which is to be expected since the limited hydrogen-bonding sites result in the increase of the contribution of the van der Waals force to form cocrystals.^61^ The hydrogen-bonding interactions (mainly O···H–N for SGD-ATP and N···H–N for the other three cocrystals) make the second or third largest contribution. For SGD-4NBA and SGD-3NBA cocrystals, the increasing hydrogen-bonding sites in the coformers lead to the increase of hydrogen-bonding interactions (mainly by providing hydrogen bond acceptors and forming O···H–N hydrogen bonds between the atoms) and the decrease of H···H contacts.

#### MEPS Analysis

**Figure 13:**
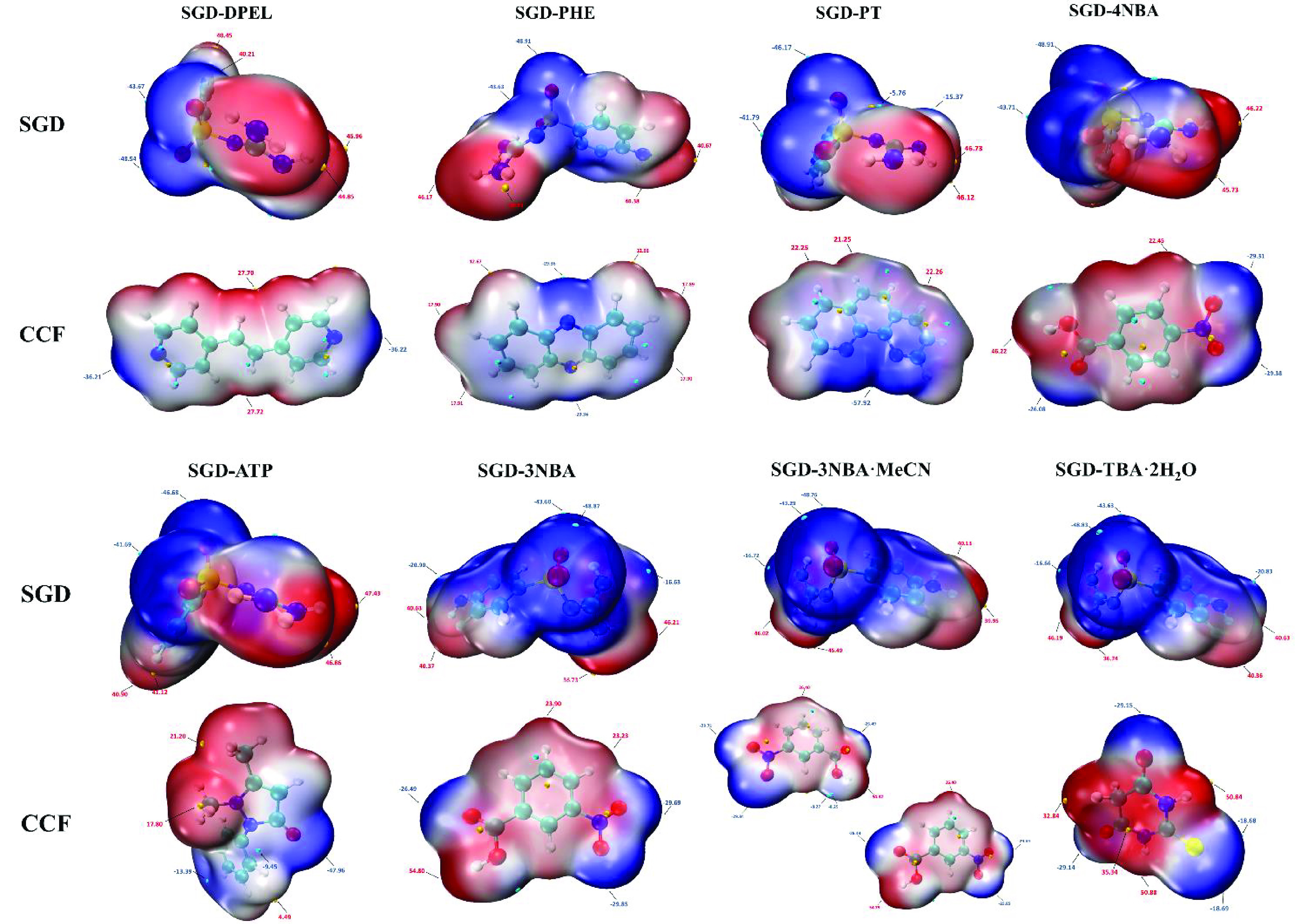
MEPS for SGD cocrystals (significant local minima and maxima of MEPS are labeled by blue and red text, respectively, and the units are kJ mol^–1^).

MEPS is critical for identifying and ranking sites for hydrogen bonding,^22^ which has been utilized as an important tool to understand and predict intermolecular interactions in the formation of cocrystals.^21,62^ The MEPS of SGD cocrystals are shown in Figure 13, where the red region represents positive potential and blue region shows negative potential.

After computation, the maximum site of the MEPS for the SGD molecule in all cocrystals is the amino groups of the guanidyl group, and the second maximum site is the amino group on the phenyl ring, while the minima values of the MEPS correspond to the two oxygen atoms from the sulfonyl group. These functional groups possess the highest hydrogen bond propensity compared to other groups and sites in the SGD molecule, while the global maxima and minima values of MEPS for the coformers in the different cocrystals vary significantly.

According to the hierarchical organization of functional group interaction theory, the main site of interaction in cocrystal formation should first occur pairwise in the minima and maxima of the MEPS.^63,64^ The formation of four SGD cocrystals (C3 in SGD-DPEL and SGD-PHE, C7 in SGD-PT, and C4 in SGD-4NBA) follows this rule, where a discrete hydrogen bond or a heterodimer occurs between the amino group from the guanidyl group in SGD and the global minima site in coformers, respectively. For the SGD-ATP cocrystal, the hydrogen bond (C1) is formed between the oxygen atom (global minima site) from ATP molecules and the amino group on the phenyl ring (second global maxima site) from SGD molecules. For SGD-3NBA, SGD-3NBA·MeCN, and SGD-TBA·H_2_O, the global maxima sites as hydrogen bond donors in coformers are engaged in the formation of hydrogen bonds with the SGD molecules where the hydrogen bond acceptors are neither the minima nor the second minima sites. Due to the complexity of multiple hydrogen bond donor and acceptor sites in both the SGD molecule and the coformer molecules, the information on hydrogen bonding ranking sites and the prediction of the most robust synthons cannot be obtained only by MEPS. To further rank the hydrogen-bonding sites and quantify the strength of hydrogen bond interactions in SGD cocrystals, QTAIM analysis was conducted.

#### QTAIM Analysis

The basic motive of QTAIM is to investigate the nature of bonding in molecular systems by exploring the charge density or electron density of molecules (ρ) and the Laplacian (∇^2^ρ) of electron density at bond critical points (BCPs), which can be utilized to distinguish between noncovalent and covalent interactions.^16,24,65^ Moreover, hydrogen-bonding interactions can also be characterized by binding energy, *E*_HBbinding_, which can be calculated using eq 1:^48^
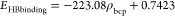
1

**Figure 14:**
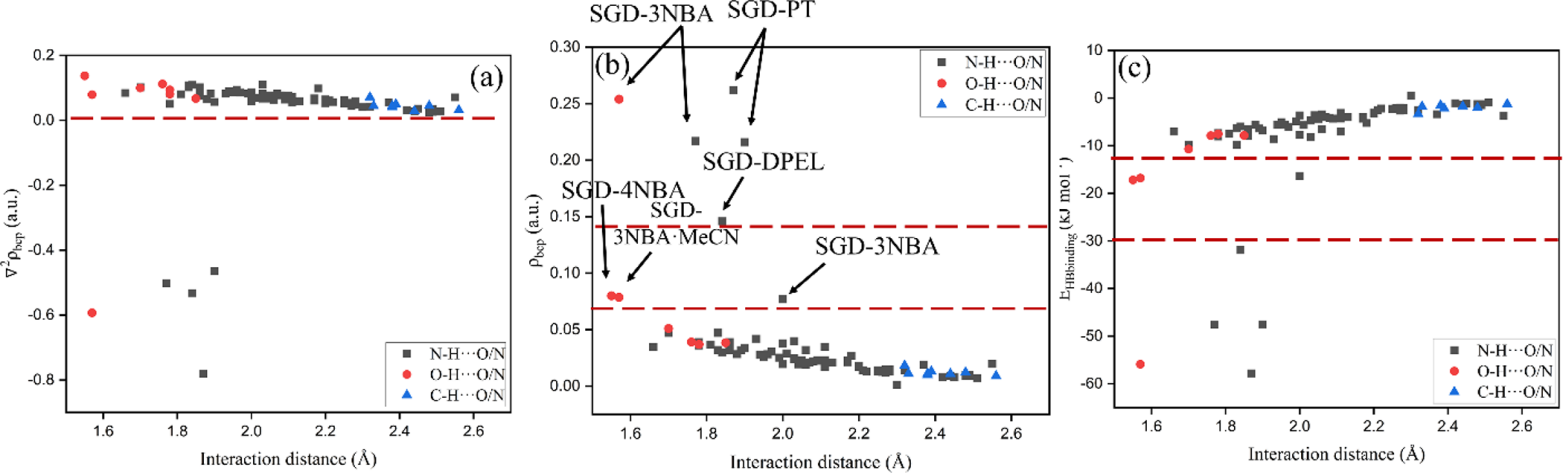
Relationship between basic QTAIM parameters: (a) ∇^2^ρ, (b) ρ_bcp_, and (c) *E*_HBbinding_ and H···A(acceptor) distances.

In the current work, QTAIM analysis was conducted to gain insight into the nature and quantify the characteristics of noncovalent interactions stabilizing the structures of the SGD cocrystals. Figure 14 and Table S7 demonstrate the relationship between basic QTAIM parameters (ρ_bcp_, *E*_HBbinding_, and ∇^2^ρ) and H···A (acceptor) distances.

As shown in Figure 14a, the ∇^2^ρ values of 83 out of 88 contacts fall into the 0–0.2 au range, suggesting that the electron density is depleted and representing noncovalent interactions, such as ionic, van der Waals, or hydrogen bonds. The ∇^2^ρ values of 5 out of 88 contacts are less than zero, which demonstrates that the density is locally concentrated, resulting in covalent bond or covalent character of interaction.^24^ Based on ∇^2^ρ and *H*_BCP_ (total electron energy density at bond critical point), Rozas and co-workers classified hydrogen bonds as follows: (i) ∇^2^ρ > 0 and *H*_BCP_ > 0 for weak hydrogen bonds, (ii) ∇^2^ρ > 0 and *H*_BCP_ < 0 for medium and strong hydrogen bonds, and (iii) ∇^2^ρ < 0 and *H*_BCP_ < 0 for very strong hydrogen bonds.^66^ Therefore, the 5 contacts are very strong hydrogen bonds.

The value of ρ_bcp_ reflects the strength of the hydrogen bonds, with low values corresponding to weak interactions, and the ρ_bcp_ value increases as the strength of the interaction increases. As shown in Table S7, the ρ_bcp_ values are in the range 0.007–0.262 au, and most hydrogen bonds are in line with the criteria proposed by Koch and Popelier,^67^ while 8 out of 88 hydrogen bonds do not correlate well with ρ_bcp_ value (Figure 14b). The upper five data points with negative values of both ∇^2^ρ and *H*_BCP_ are very strong hydrogen bonds, and three data points with relatively higher ρ_bcp_ values lie in the middle region, representing the medium-strong hydrogen bonds, which are also supported by the positive ∇^2^ρ values and negative *H*_BCP_ values of these interactions. The value of *E*_HBbinding_ is another approach to quantify the strength of hydrogen bonds: the lower *E*_HBbinding_ value corresponding to stronger interactions, and vice versa (Figure 14c).

All the medium-strong and very strong hydrogen bonds are involved in the formation of heterosynthons between the guanidyl group of SGD and the coformers, except one interaction in SGD-PT which forms the homosynthon between two SGD molecules. More specifically, for SGD-3NBA, very strong and medium-strong hydrogen bonds are involved in the formation of two *R*_2_^2^(8) motifs (C4 and C6) between SGD and two 3NBA molecules. The two very strong hydrogen bonds in the SGD-PT cocrystal are engaged in the formation of an *R*_2_^2^(8) homosynthon (A1) between two SGD molecules and an *R*_2_^2^(9) heterosynthon (C7) between SGD and PT molecules, respectively. The very strong hydrogen bond in SGD-DPEL and medium-strong hydrogen bond in SGD-PHE form a discrete synthon (C3) between SGD and coformers, respectively. Both of the medium-strong hydrogen bonds in SGD-4NBA and SGD-3NBA·MeCN are found in the structures of the *R*_2_^2^(8) heterosynthons (C4). This reveals that it is both experimentally and computationally favorable for these cocrystals to form.

**Figure 15:**
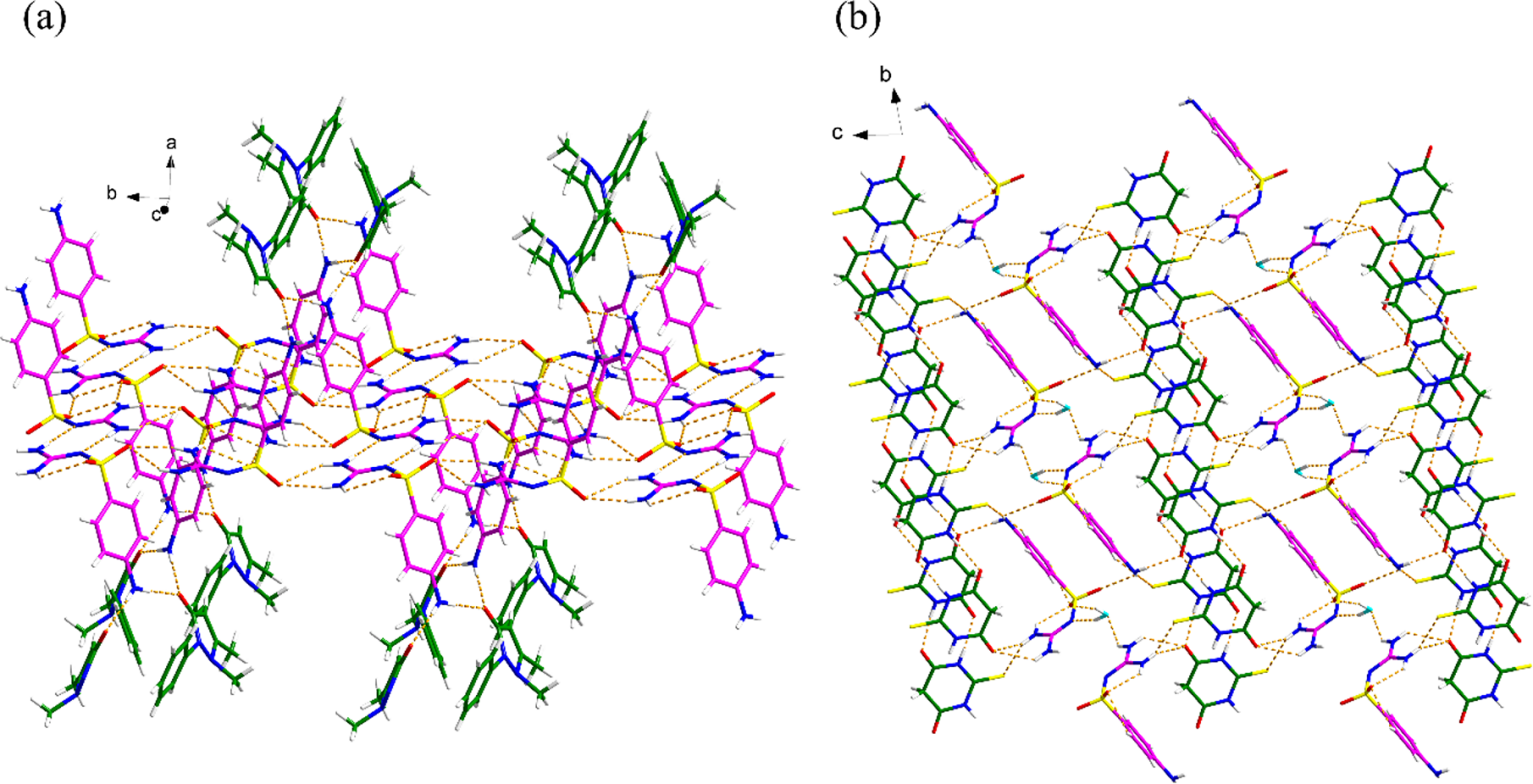
Crystal packing of (a) SGD-ATP and (b) SGD-TBA·2H_2_O (pink is SGD, green is coformer, and blue is H_2_O).

According to Table S7, no strong hydrogen bonds can be found in the SGD-ATP cocrystal, and the first three relatively stronger hydrogen bond interactions occur between SGD molecules instead of between SGD and ATP molecules with the strength of hydrogen bonds in the following order: C1 < A2 < B1< A1. For SGD-TBA·2H_2_O, the first six relatively stronger hydrogen bonds are between two TBA molecules or between SGD and H_2_O molecules, indicating that the propensity of forming hydrogen bonds between SGD and TBA is weaker. From the crystal packing (Figure 15) there are more hydrogen bond interactions between two SGD molecules or between two coformer molecules than there are between SGD and coformer molecules.

**Figure 16:**
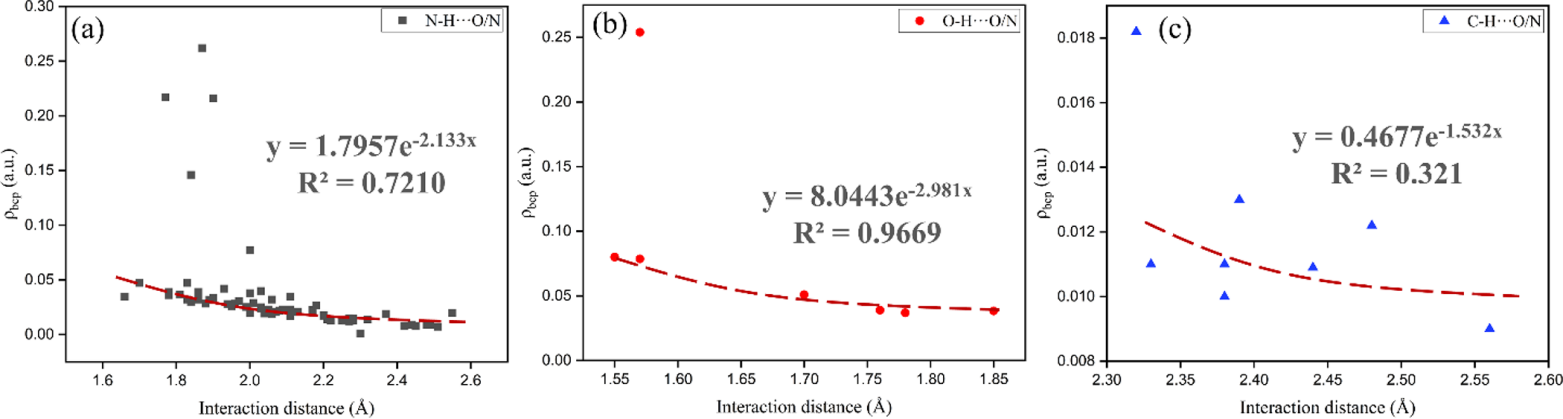
Relationship between the interaction distance and the electron density (ρ_bcp_) of (a) N–H···O/N, (b) O–H···O/N, and (c) nonconventional hydrogen bond interactions (C–H···O/N) (strong and medium-strong hydrogen bonds ignored for the regression).

Current study on hydrogen bonds within cocrystals has focused the most attention upon the ability of functional groups to form conventional hydrogen bonds of the O–H···O or N–H···O type because these traditional hydrogen bonds can be expected to represent the strongest sort of interaction, which are considered the main driving forces for the cocrystal formation. As mentioned above, for the studied cocrystals, all of the robust hydrogen bonds are conventional hydrogen bonds. For N–H···O/N hydrogen bonds, an exponential dependence between the distance and ρ_bcp_ can be found, with *R*^2^ factors of 0.7210 (Figure 16a). The exponential correlation (*R*^2^ = 0.9669) is also found between the ρ_bcp_ and H···A distances for the ···H···O/N contacts (Figure 16b), revealing that ρ_bcp_ is a good descriptor of the strength of weak conventional hydrogen bonds. Nonconventional hydrogen bonds such as C–H···O/N hydrogen bonds also play a non-negligible role in the stabilization of 3D structure of the SGD cocrystals. However, no significant correlation between the ρ_bcp_ and H···A distances for C–H···O/N contacts can be found (*R*^2^ = 0.3210), which may be due to the relatively low strength of this type of hydrogen bond (Figure 16c).

**Figure 17:**
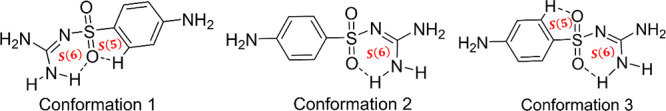
Conformations of the SGD molecule existing in different crystalline forms.

Some work reported that the QTAIM analysis is not able to detect all expected weak noncovalent interactions,^68,69^ which is also found in this work. As shown in Figure 17, SGD adopts conformation 1 in SGD·H_2_O, SGD-3NBA, SGD-PHE, SGD-PT, and SGD-TBA·2H_2_O, generating S(6) and S(5) rings. In SGD-3NBA·MeCN and SGD-ATP, only one N–H···O intramolecular hydrogen bond interaction forming an S(6) ring can be found in SGD conformation 2. For conformation 3 in SGD, SGD-4NBA, and SGD-DPEL, two oxygen atoms from the sulfonyl group are involved in the construction of S(6) and S(5) rings. Notably, the bond angles of all hydrogen bonds forming S(5) rings in SGD molecules are less than 110°, which are not in the common range 120–180°.^70^ However, these angles fall within the range of geometric limits provided by the IUPAC definition of the hydrogen bond.^71^ The bond critical points of the C–H···O intramolecular hydrogen bonds involving the formation of an S(5) ring in the crystal structures were not found after optimization. This suggests that conformations 1 and 3 may not be the preferable molecular geometry of SGD, and the conformation of the SGD moiety in those cocrystals is changed with the breakage of the C–H···O intramolecular hydrogen bonds after optimization, resulting in no critical points for those intramolecular hydrogen bonds.

## Conclusions

This study reports the synthesis and characterization of five novel cocrystals of SGD with four coformers (DPEL, PHE, 4NBA, and 3NBA). A detailed crystal structural analysis was performed, and computational calculations including Hirshfeld surface, MEPS, and QTAIM analyses have been applied to investigate the different hydrogen-bonding interactions within all SGD cocrystals. Hirshfeld surface analysis revealed that the increasing hydrogen donor/acceptor sites in the coformers lead to the increase of hydrogen-bonding interactions and a decrease of H···H contacts in SGD cocrystals and vice versa. The main site of interaction in the formation of four out of eight cocrystals first occurred pairwise in the minima and maxima of the MEPS; however, the prediction of the most robust synthons cannot be obtained correctly by MEPS due to the complexity of the various hydrogen bond donor and acceptor sites in both SGD and the coformer molecules. QTAIM analysis was conducted as a complementary tool to quantify the strength of hydrogen bond interactions, illustrating that all of the medium-strong and very strong hydrogen bonds are involved in the formation of heterosynthons between SGD and coformers. This indicates that the formation of those SGD cocrystals is both experimentally and computationally favorable. In this study, QTAIM analysis showed superiority over MEPS analysis to obtain a comprehensive understanding of hydrogen bond interactions when there are multiple hydrogen bond donor and acceptor sites in cocrystallizing components.

## Supporting Information Available

DSC traces, IR spectra, PXRD patterns, ellipsoid plots, table of crystallographic parameters of five obtained SGD cocrystals and SGD-ATP cocrystal, and selected topological parameters of electron density distribution [au] for SGD cocrystals (PDF)
