# Supplementary material for: Experimental and Theoretical Investigation of Hydrogen-Bonding Interactions in Cocrystals of Sulfaguanidine
Source: Cryst Growth Des. 2023 Mar 1;23(4):2306–20. doi: 10.1021/acs.cgd.2c01337 (PMC10080660; doi:10.1021/acs.cgd.2c01337)
Supplement: Supplementary file 1 — cg2c01337_si_001.pdf [file cg2c01337_si_001.pdf]

Supporting Information

Experimental and Theoretical Investigation of Hydrogen Bonding Interactions in Cocrystals of Sulfaguanidine

Shan Huang, [†,‡] *Vinay K. R. Cheemarla,* [†] *Davide Tiana,* [†] *and Simon E. Lawrence*[†,‡,]*

[†]School of Chemistry, Synthesis and Solid State Pharmaceutical Centre, University College Cork, Cork T12 K8AF, Ireland

[‡]Analytical and Biological Chemistry Research Facility, University College Cork, Cork T12 K8AF, Ireland

Contents

DSC traces of SGD, coformers and cocrystals	2
IR spectra of SGD, coformers and cocrystals	3
PXRD patterns of SGD, coformers and cocrystals	5
Ellipsoid plots of SGD cocrystals	6
Hydrogen bond and π–π interaction geometries in the SGD cocrystals	8
Selected topological parameters of electron density distribution for cocrystals	14
References	19

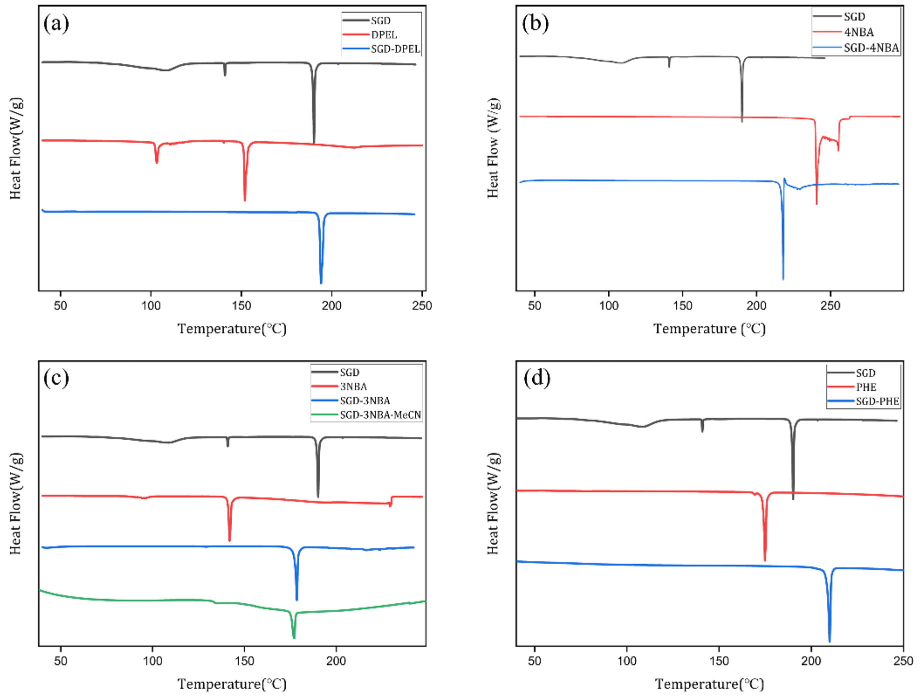

Figure S1. DSC traces of (a) SGD·H_2O (black), DPEL (red) and SGD-DPEL cocrystal (blue); (b) SGD·H_2O (black), 4NBA (red) and SGD-4NBA cocrystal (blue); (c) SGD·H_2O (black), 3NBA (red), SGD-3NBA cocrystal (blue) and SGD-3NBA·MeCN cocrystal solvate (green); and (d) SGD·H_2O (black), PHE (red) and SGD-PHE cocrystal (blue).

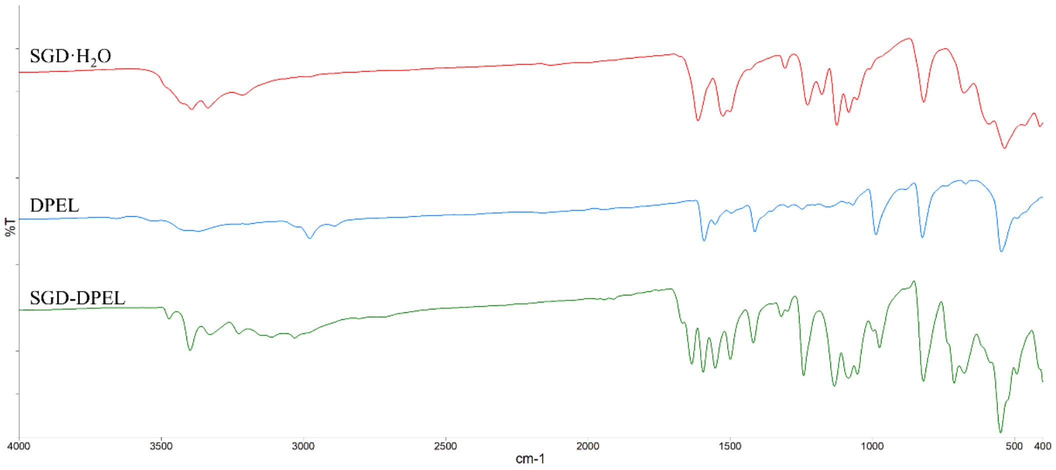

Figure S2. IR spectra of (a) SGD·H_2O, DPEL and SGD-DPEL cocrystal.

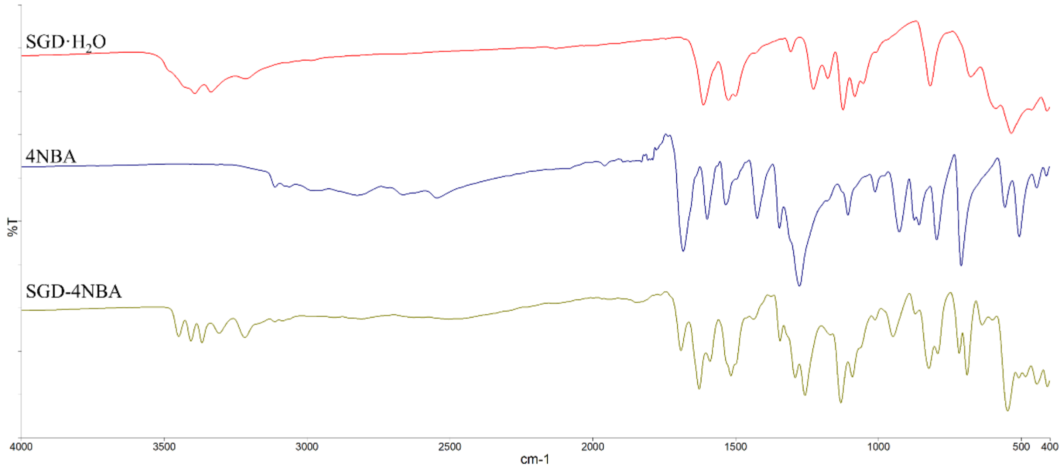

Figure S3. IR spectra of SGD·H_2O, 4NBA and SGD-4NBA cocrystal.

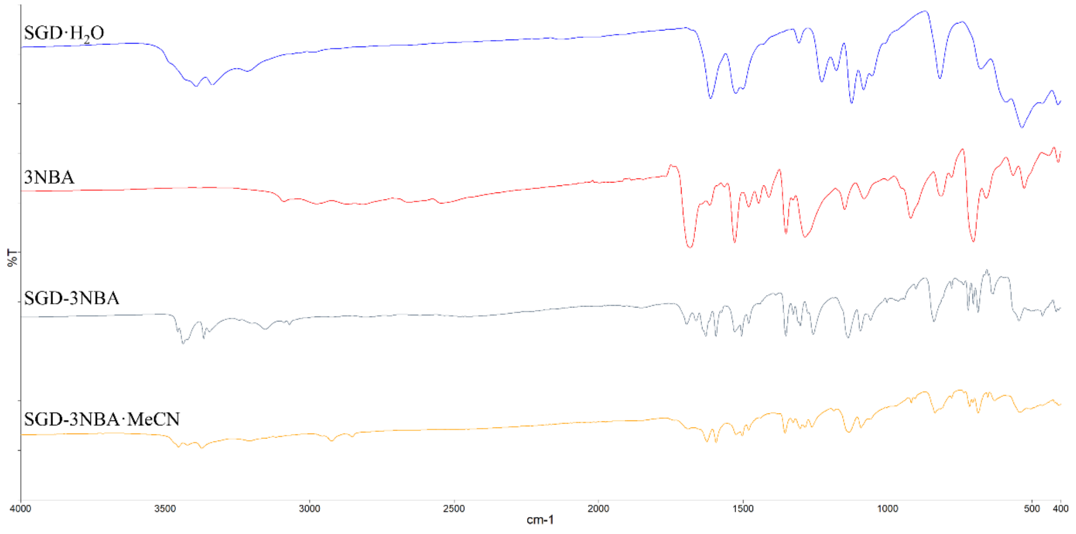

Figure S4. IR spectra of SGD·H_2O, 3NBA, SGD-3NBA cocrystal and SGD-3NBA·MeCN cocrystal solvate.

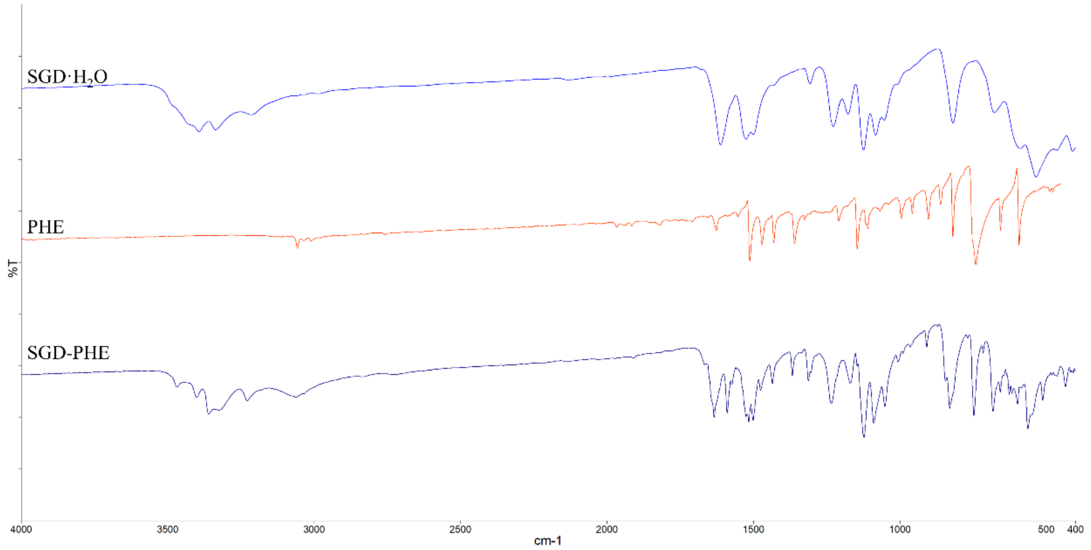

Figure S5. IR spectra of SGD·H_2O, PHE and SGD-PHE cocrystal.

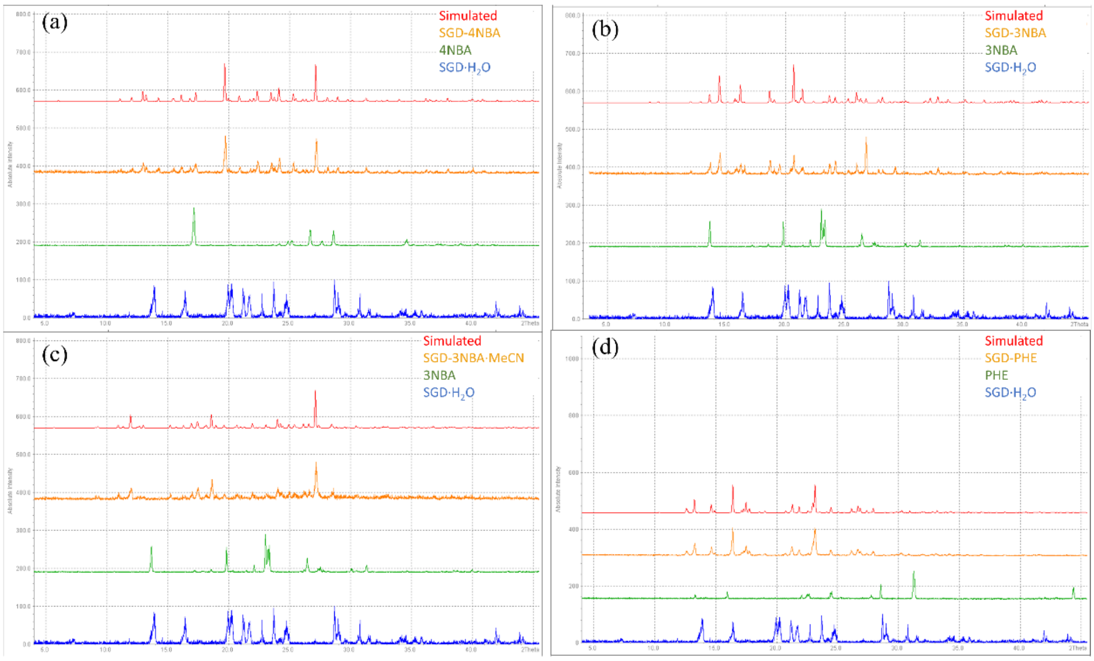

Figure S6. PXRD patterns of (a) SGD·H_2O (blue), 4NBA (green), SGD-4NBA (orange) and simulated pattern from the crystal structure (red); (b) PXRD patterns of SGD·H_2O (blue), 3NBA (green), SGD-3NBA (orange) and simulated pattern from the crystal structure (red); (c) PXRD patterns of SGD·H_2O (blue), 3NBA (green), SGD-3NBA·MeCN (orange) and simulated pattern from the crystal structure (red); (d) PXRD patterns of SGD·H_2O (blue), PHE (green), SGD-PHE (orange) and simulated pattern from the crystal structure (red).

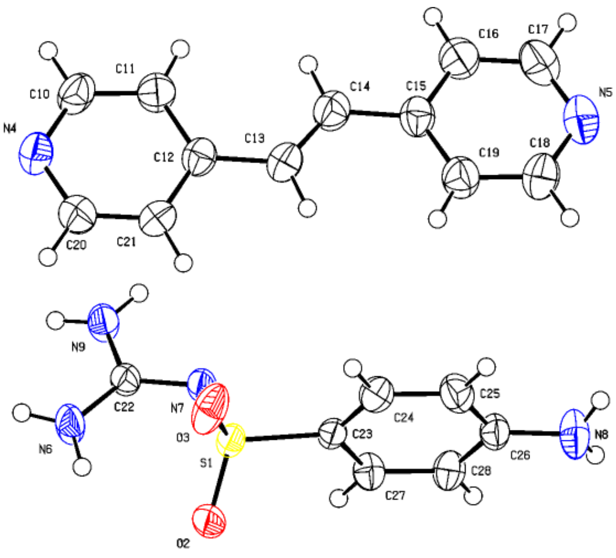
C16
C17
C11
C14
C15
N5
C10
N4
C13
C12
C19
C18
C20
C21
N9
C25
C24
C22
N7
N8
O3
C23
C26
N6
S1
C28
C27
O2

(a)

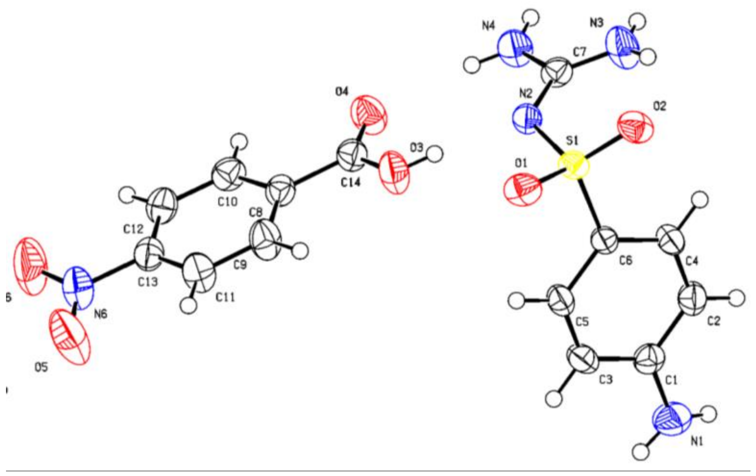
N4
N3
C7
O4
N2
O2
O3
S1
C14
O1
C10
C8
C12
C6
C4
C9
C13
C11
C2
N6
C5
O5
C3
C1
N1

(b)

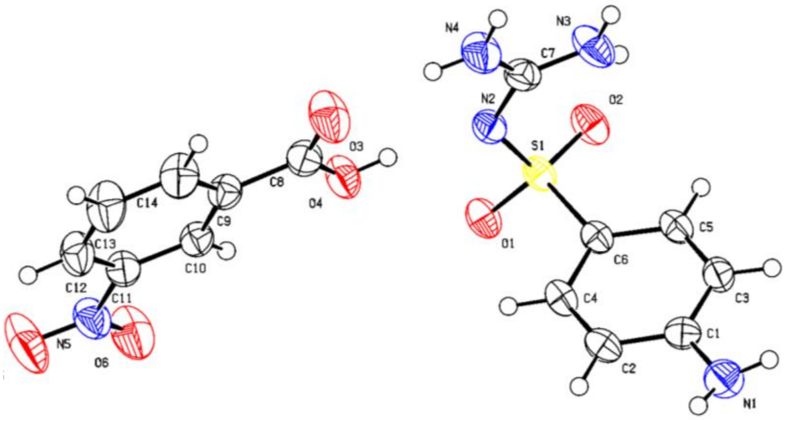
N4
N3
C7
N2
O2
S1
O3
C8
O4
C14
C9
C5
C13
O1
C6
C12
C11
C10
C3
C4
N5
O6
C1
C2
N1

(c)

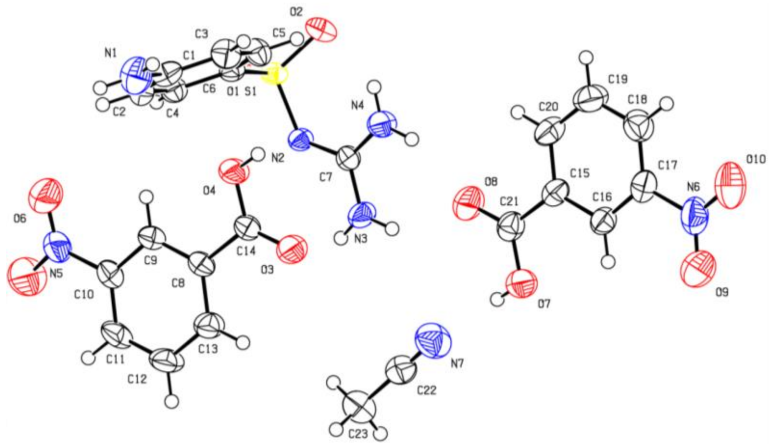

(d)

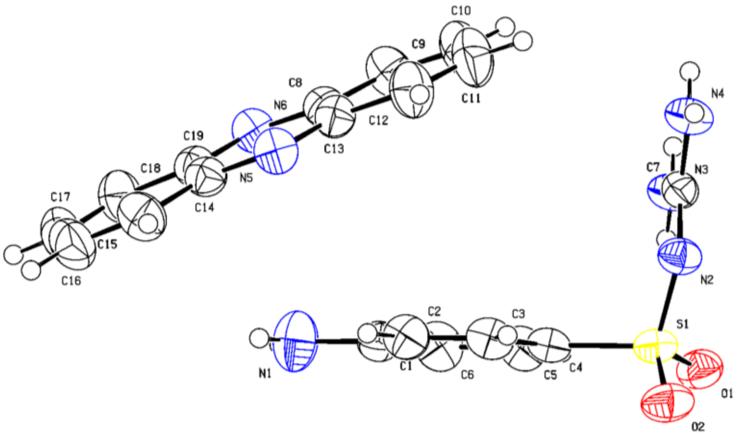

(e)

Figure S7. Ellipsoid plot of (a) SGD-DPEL, (b) SGD-4NBA, (c) SGD-3NBA, (d) SGGD-3NBA·MeCN and (e) SGD-PHE.

Table S1. Hydrogen bond and π–π interaction geometries (Å, °) in the SGD-DPEL cocrystal [a]

	Type	D-H···A	D-H	H···A	D···A	D-H···A	ARU (J)
1		N3-H11···N1	0.852(19)	2.065(19)	2.914(2)	175.4(18)	[1455.02]
2		N5-H12···N2	0.856(15)	2.28(2)	3.049(2)	150(2)	[1656.02]
3		N6-H14···N4	0.858(18)	2.134(18)	2.9912(18)	176.6(18)	[2666.01]
4		N5-H15···O2	0.842(19)	2.22(2)	3.016(2)	157.8(17)	[1655.01]
5	Intra	N3-H20··O1	0.851(11)	2.419(18)	2.9941(19)	125.5(15)	
6		N3-H20···O1	0.851(11)	2.201(13)	2.9151(16)	141.4(18)	[2656.01]
7		C12-H5···O2	0.93	2.58	3.4553(19)	157	[2666.01]
8	Intra	C15-H17···O2	0.93	2.55	2.917(2)	104	

Cg(I)	Cg(J)	Cg-Cg	Interplanar distance	Dihedral Angle	Beta	ARU (J)
2	3	3.8818(11)	3.3836(7)	6.22(8)	31.2	[1655.02]

[a] Symmetry codes: [2656.] = [2_656] = 1-x, -y, 1-z; [1455.] = [1_455] = -1+x, y, z; [2666.] = [2_666] = 1-x, 1-y, 1-z; [1655.] = [1_655] = 1+x, y, z; [1656.] = [1_656] = 1+x, y, 1+z. Cg2 represents the centroid of N1-C1-C2-C3-C12-C11 and Cg3 represents the centroid of N2-C8-C7-C6-C10-C9. Cg(I) = plane number I; Cg–Cg = distance between ring centroids (Ang.). Beta is the displacement angle between the ring normal of plane I and the centroid vector.

Table S2. Hydrogen bond and π–π interaction geometries (Å, °) in the SGD-4NBA cocrystal [a]

	Type	D-H···A	D-H	H···A	D···A	D-H···A	ARU (J)
1		N3-H13···O1	0.865(13)	1.962(13)	2.8155(16)	168.8(14)	[1565.01]
2	Intra	N3-H14···O2	0.800(15)	2.151(14)	2.7601(17)	133.2(13)	
3		O3-H15···N2	0.836(15)	1.854(15)	2.6859(15)	173.2(14)	[1555.01]
4		N4-H16···O1	0.842(13)	2.571(13)	3.2482(17)	138.4(11)	[1565.01]
5		N4-H17···O4	0.872(13)	2.145(13)	3.0093(17)	171.2(12)	[1555.02]
6		N1-H18···O2	0.871(13)	2.411(12)	3.1442(16)	142.1(11)	[4564.01]
7	Intra	C5-H5···O1	0.93	2.57	2.9272(15)	103	
8	Intra	C9-H9···O3	0.93	2.4	2.7166(18)	100	

[a] Symmetry codes: [4564.] = [6_564] = x, 1-y, -1/2+z; [1565.] = [1_565] = x, 1+y, z.

Table S3. Hydrogen bond and π–π interaction geometries (Å, °) in the SGD-3NBA cocrystal [a]

	Type	D-H···A	D-H	H···A	D···A	D-H···A	ARU (J)
**1**		N1-H1···O1	0.894(10)	2.465(10)	3.2091(11)	141.0(11)	[6545.01]
**2**		N1-H1···O4	0.894(10)	2.585(11)	3.2905(12)	136.4(8)'	[6545.02]
**3**		N3-H15···O6	0.851(10)	2.249(10)	3.0998(11)	177.7(8)	[1545.02]
**4**	Intra	N3-H16···O2	0.855(11)	2.227(10)	2.8558(11)	130.3(8)	
**5**		N3-H16···O2	0.855(11)	2.270(10)	3.0144(12)	145.5(9)'	[7555.01]
**6**		N4-H17···O5	0.838(10)	2.196(10)	3.0285(12)	173.0(12)	[1545.02]
**7**		N4-H18···O3	0.905(10)	1.959(10)	2.8588(11)	173.0(10)	[1555.02]
**8**		O4-H19···N2	0.847(11)	1.842(12)	2.6835(10)	172.2(15)	[1555.01]
**9**		N1-H20···O1	0.849(10)	2.475(10)	3.2707(11)	156.6(12)	[4565.01]
**10**		C2-H2···O2	0.93	2.51	3.4397(10)	173	[4565.01]
**11**	Intra	C5-H2···O2	0.93	2.58	2.9415(12)	103	
**12**		C10-H10···N1	0.93	2.62	3.5427(14)	172	[6555.01]

[a] Symmetry codes: [6555.] = [7_555] = 1/2-x, 1/2+y, 1/2-z; [7555.] = [4_555] = 1/2-x, 1/2-y, -z; [6545.] = [7_545] = 1/2-x, -1/2+y ,1/2-z; [1545.] = [1_545] = x, -1+y, z; [4565.] = [6_565] = x, 1-y, 1/2+z.

Table S4. Hydrogen bond and π–π interaction geometries (Å, °) in the SGD-3NBA·MeCN [a]

	Type	D-H···A	D-H	H···A	D···A	D-H···A	ARU (J)
1		N1-H1A···O1	0.86	2.57	3.354(3)	153	[1655.01]
2		N3-H3A···O8	0.86	2.35	3.086(3)	144	[1555.03]
3		N3-H3B···O3	0.86	2.01	2.870(3)	174	[1455.02]
4		N4-H4A···O8	0.86	2.03	2.848(3)	158	[1555.03]
5	Intra	N4-H4B···O2	0.86	2.19	2.812(3)	129	
6		N4-H4B···O2	0.86	2.33	2.939(3)	129	[2676.01]
7		O4-H4C···N2	0.82	1.87	2.681(3)	170	[1655.01]
8		O7-H7···N7	0.82	2.01	2.829(4)	174	[1555.04]
9		C3-H3···O1	0.93	2.54	3.385(3)	151	[1655.01]
10		C11-H11···O5	0.93	2.53	3.259(4)	136	[2667.02]
11		C19-H19···O1	0.93	2.56	3.313(4)	138	[2676.01]
12		C23-H23C···O3	0.96	2.53	3.425(4)	155	[1455.02]
**Cg(I)**	**Cg(J)**	**Cg-Cg**	**Interplanar distance**		**Dihedral Angle**	**Beta**	**ARU (J)**
2	3	3.8615(17)	3.4490(11)		3.98(14)	23	[2666.03]

[a] Symmetry codes: [1455.] = [1_455] = -1+x, y, z; [2676.] = [2_676] = 1-x, 2-y, 1-z; [1655.] = [1_655] = 1+x, y, z; [2667.] = [2_667] = 1-x, 1-y, 2-z; [2666] = 1-x, 1-y, 1-z. Cg2 represents the centroid of C8–C13 and Cg2 represents the centroid of C15–C20. Cg(I) = plane number I; Cg–Cg = distance between ring centroids (Ang.). Beta is the displacement angle between the ring normal of plane I and the centroid vector.

Table S5. Hydrogen bond and π–π interaction geometries (Å, °) in the SGD-PHE cocrystal [a]

	Type	D-H···A	D-H	H···A	D···A	D-H···A	ARU (J)
1		N1-H1A···O2	0.86	2.46	3.220(2)	148	[1455.01]
2		N3-H3A···O2	0.86	2.14	2.985(2)	169	[2655.01]
3	Intra	N3-H3B···O1	0.86	2.29	2.8953(19)	128	
4		N3-H3B···N5	0.86	2.62	3.313(2)	139	[4564.02]
5		N4-H4A···O1	0.86	2.08	2.930(2)	169	[2655.01]
6		N4-H4B···N6	0.86	2.08	2.936(2)	175	[1655.02]
7	Intra	C5-H5···O1	0.93	2.53	2.903(2)	105	

Cg(I)	Cg(J)	Cg-Cg	Interplanar distance	Dihedral Angle	Beta	ARU (J)
2	3	4.0823(12)	3.6008(7)	1.96(9)	28.6	[3576.02]

[a] Symmetry codes: [4564.] = [4_575] = x, 3/2-y, -1/2+z; [1455.] = [1_455] = -1+x, y, z; [2655.] = [2_655] = 1-x, 1/2+y, 1/2-z; [1655.] = [1_655] = 1+x, y, z; [3576] = -x, 2-y, 1-z. Cg2 represents the centroid of N5-C13-C8-N6-C19-C14 and Cg3 represents the centroid of C8-C9-C10-C12-C12-C13. Cg(I) = plane number I; Cg–Cg = distance between ring centroids (Ang.). Beta is the displacement angle between the ring normal of plane I and the centroid vector.

Table S6. Hydrogen bond and π–π interaction geometries (Å, °) in the SGD-ATP cocrystal [1 a]

	Type	D-H···A	D-H	H···A	D···A	D-H···A	ARU (J)
1		N1-H3···O3	0.83(7)	2.24(7)	3.056(9)	167(7)	[2756.02]
2		N1-H5···O3	0.77(7)	2.38(7)	3.145(9)	175(8)	[3756.02]
3		N3-H6···N2	0.86(7)	2.11(7)	2.974(7)	176(9)	[3656.01]
4		N3-H7···O1	0.85(7)	2.57(7)	3.279(7)	142(6)	[2645.01]
5		N4-H8··O1	0.82(7)	2.15(7)	2.937(7)	161(7)	[2645.01]
6	Intra	N4-H9···O2	0.75(8)	2.43(7)	2.832(7)	116(7)	
7		N4-H9···O2	0.75(8)	2.20(8)	2.912(7)	160(7)	[3655.01]

Cg(I)	Cg(J)	Cg-Cg	Interplanar distance	Dihedral Angle	Beta	ARU (J)
2	3	4.025(5)	3.341(3)	16.4(4)	22.1	[4544.02]
3	2	4.026(5)	3.731(4)	16.4(4)	33.9	[4545.02]

[a] Symmetry codes: [3656.] = [3_656] = 1-x, -y, 1-z; [3655.] = [3_655] = 1-x, -y, -z; [2756.] = [2_756] = 2-x, 1/2+y, 3/2-z; [3756.] = [3_756] = 2-x, -y, 1-z; [2645.] = [2_645] = 1-x, -1/2+y, 1/2-z; [4544] = x, -1/2-y, -1/2+z; [4545] = x, -1/2-y, 1/2+z. Cg2 represents the centroid of N5-N6-C10-C9-C8 and Cg3 represents the centroid of C13-C14-C15-C16-C17-C18. Cg(I) = plane number I; Cg–Cg = distance between ring centroids (Ang.). Beta is the displacement angle between the ring normal of plane I and the centroid vector.

Table S7. Selected topological parameters of electron density distribution [au] for SGD crystals

	Type	D-H···A	H···A [a]	ρ bcp (au)	$\nabla^2 \rho_{bcp}$ (au)	G_{bcp} kJ mol^{-1}	V_{bcp} kJ mol^{-1}	H_{bcp} kJ mol^{-1}	$E_{binding}$ kJ mol^{-1}
SGD·H_2O[2]		O3-H4···O2	1.78	0.037	0.0939	30.975	-0.462	30.513	-7.530
		O3-H3···O1	1.76	0.039	0.112	33.338	6.851	40.189	-7.870
		N3-H11···O3	1.94	0.028	0.0902	19.924	19.346	39.270	-5.590
		N3-H10···O3	2.04	0.022	0.0769	13.414	23.599	37.013	-4.260
		N4-H2···O2	2.11	0.017	0.0617	8.636	23.179	31.815	-3.100
		N4-H12···O3	2.21	0.014	0.0611	6.458	27.300	33.758	-2.480
	Intra	N4-H2···O1	2.28	0.0149	0.0584	6.799	24.728	31.526	-2.580
SGD-DPEL		N3-H11···N1	1.84	0.146	-0.532	304.500	-960.750	-656.250	-480.375
		N6-H14···N4	1.93	0.042	0.083	38.063	-21.236	16.826	-10.618
		N5-H12···N2	2.06	0.032	0.063	24.728	-8.111	16.616	-4.056
		N5-H15···O2	2.03	0.025	0.067	15.619	12.784	28.403	6.392
		N3-H20···O1	2.06	0.020	0.077	11.183	27.825	39.008	13.913
	Intra	N3-H20···O1	2.37	0.019	0.056	9.739	17.430	27.169	8.715
		C12-H5···O2	2.39	0.013	0.050	5.381	22.050	27.431	11.025
SGD-4NBA		O3-H15···N2	1.57	0.0786	0.0793	108.675	-165.375	-56.700	-16.800
		N3-H13···O1	1.78	0.0361	0.0504	29.663	-26.513	3.150	-7.320
		N4-H17···O4	1.89	0.0319	0.0823	24.150	5.670	29.820	-6.370
	Intra	N3-H14···O2	1.97	0.0306	0.0852	22.628	10.658	33.285	-6.090
		N1-H18···O2	2.18	0.0268	0.0986	18.086	28.613	46.699	-5.230

		N4-H16···O1	2.51	0.00718	0.0268	2.013	13.545	15.558	-0.859
SGD-3NBA		O4-H19···N2	1.57	0.254	-0.593	769.125	-1924.125	-1155.000	-55.900
		N4-H18···O3	1.77	0.217	-0.502	590.625	-1509.375	-918.750	-47.600
		N4-H17···O5	2.00	0.077	0.0858	105.000	-153.825	-48.825	-16.400
	Intra	N3-H16···O2	2.17	0.0226	0.0607	13.624	12.548	26.171	-4.300
		N3-H15···O6	2.03	0.0399	0.111	35.175	2.544	37.719	-8.150
		N3-H16···O2	2.17	0.0213	0.0677	12.285	19.845	32.130	-4.000
		N1-H1···O1	2.27	0.0122	0.0441	4.909	4.909	9.818	-1.990
		N1-H20···O1	2.25	0.0139	0.0625	6.038	28.875	34.913	-2.360
		C2-H2···O2	2.32	0.0182	0.071	9.476	27.563	37.039	-3.320
		N1-H1···O4	2.49	0.01	0.0263	3.491	10.264	13.755	-1.490
		C10-H10···N1	2.48	0.0122	0.0449	4.856	19.766	24.623	-1.980
SGD-3NBA·MeCN		O4-H4C···N2	1.55	0.08	0.137	112.613	-135.188	-22.575	-17.200
		O7-H7···N7	1.70	0.051	0.0996	53.288	-41.213	12.075	-10.700
		N3-H3B···O3	1.78	0.039	0.0842	34.388	-13.571	20.816	-8.050
		N4-H4A···O8	1.83	0.032	0.108	23.861	23.048	46.909	-6.320
	Intra	N4-H4B···O2	2.08	0.022	0.0769	13.046	24.386	37.433	-4.170
		N4-H4B···O2	2.22	0.013	0.0566	5.513	26.093	31.605	-2.190
		N3-H3A···O8	2.25	0.013	0.051	5.618	22.208	27.825	-2.220
		C23-H23C···O3	2.33	0.011	0.0446	4.174	20.921	25.095	-1.740
		C11-H11···O5	3.00	0.002	0.0085	0.243	5.119	5.361	0.293

		C3-H3···O1	2.38	0.01	0.041	3.465	19.950	23.415	-1.480
		C19-H19···O1	2.38	0.011	0.0447	3.780	21.788	25.568	-1.590
		N1-H1A···O1	2.42	0.008	0.0309	2.449	15.356	17.805	-1.060
SGD[3]		N3-H8···O4	1.84	0.030	0.109	21.788	28.147	49.935	-5.942
		N3-H7···N8	2.01	0.029	0.070	20.181	5.759	25.940	-5.642
	Intra	N6-H15···O3	2.03	0.024	0.082	15.041	23.625	38.666	-4.612
		N4-H10···O3	2.06	0.019	0.069	9.765	25.751	35.516	-3.391
	Intra	N2-H5···O1	2.07	0.021	0.083	13.140	28.481	41.621	-3.994
		N7-H18···N1	2.09	0.023	0.064	13.650	14.385	28.035	-4.299
		N6-H16···N4	2.11	0.023	0.055	13.571	9.004	22.575	-4.277
		N8-H19···N5	2.13	0.021	0.059	12.180	14.464	26.644	-3.965
		N7-H17···N5	2.20	0.018	0.053	9.240	15.960	25.200	-3.251
		N2-H6···O2	2.30	0.001	0.041	3.431	20.213	23.643	0.522
		N4-H9···O2	2.32	0.014	0.042	4.383	18.579	22.961	-2.489
		N8-H20···O2	2.44	0.009	0.028	3.045	12.338	15.383	-1.306
		N6-H15···O1	2.45	0.008	0.036	2.651	18.113	20.764	-1.149
		C5-H3···O3	2.56	0.009	0.032	2.861	15.041	17.903	-1.239
SGD-PHE		N4-H4A···O1	1.86	0.032	0.102	24.649	17.955	42.604	-6.460
		N4-H4B···N6	1.86	0.039	0.075	33.600	-18.139	15.461	-7.930
		N3-H3A···O2	1.95	0.026	0.086	17.010	22.076	39.086	-5.020
	Intra	N3-H3B···O1	2.27	0.015	0.054	6.983	21.105	28.088	-2.640

		N1-H1A···O2	2.28	0.013	0.048	5.119	20.974	26.093	-2.050
		N3-H3B···N5	2.48	0.010	0.031	3.754	12.653	16.406	-1.590
SGD-PT		N1-H6··N4	1.90	0.216	-0.464	588.000	-1480.500	-892.500	-47.500
	Intra	N3-H7···O2	2.00	0.0379	0.059	32.288	-25.778	6.510	-7.710
		N1-H5···N5	1.87	0.262	-0.781	811.125	-2131.500	-1320.375	-57.800
		N2-H9···O2	1.99	0.0252	0.0827	16.380	21.499	37.879	-4.890
		N2-H8···O1	2.05	0.023	0.067	13.965	16.013	29.978	-4.380
		N3-H10···N6	2.11	0.0348	0.073	28.088	-8.111	19.976	-7.030
		N1-H5···N6	2.55	0.0198	0.0707	10.920	24.544	35.464	-3.680
		C12-H12···O1	2.44	0.0109	0.0288	4.043	10.841	14.884	-1.690
SGD-ATP[1]		N3-H6···N2	1.90	0.0339	0.0569	26.775	-16.170	10.605	-6.820
		N4-H8···O1	1.88	0.0284	0.0654	19.898	3.124	23.021	-5.590
		N4-H9···O2	2.04	0.0193	0.0767	10.500	29.400	39.900	-3.570
		N1-H4···O3	2.00	0.0195	0.0711	10.684	25.331	36.015	-3.620
		N1-H5···O3	2.10	0.0225	0.0759	13.519	22.759	36.278	-4.280
	Intra	N4-H9···O2	2.20	0.0171	0.0641	8.531	25.016	33.548	-3.060
		N3-H7···O1	2.48	0.00896	0.0244	2.914	10.185	13.099	-1.260
SGD-TBA·2H_2O		N5-H11···O5	1.83	0.0472	0.102	46.463	25.804	72.266	-9.790
		O7-H19···N1	1.85	0.0384	0.067	33.075	-21.998	11.078	-7.830
		N6-H12···O6	1.81	0.0368	0.0806	30.713	-8.505	22.208	-7.470
		N8-H16···O4	1.70	0.0472	0.102	46.463	-25.804	20.659	-9.790

		O7-H20···O2	1.78	0.0369	0.0808	30.975	-8.689	22.286	-7.500
		N7-H15···O3	1.66	0.0346	0.0845	27.563	0.185	27.748	-6.970
		N4-H9···O5	3.65	0.00898	0.0351	2.914	17.168	20.081	-1.260
	Intra	N3-H4···O2	1.96	0.0283	0.0945	19.793	22.418	42.210	-5.570
		N2-H2···O7	1.86	0.0335	0.0967	26.250	10.894	37.144	-6.740
		N3-H3···O4	2.26	0.0126	0.0512	5.145	23.310	28.455	-2.070
		N2-H1···O4	2.49	0.00898	0.0351	2.914	17.168	20.081	-1.260
		C14-H17···O3	2.39	0.0106	0.0419	3.833	19.819	23.651	-1.620
		N4-H10···S3	2.74	0.00875	0.0274	2.809	12.416	15.225	-1.210
		N4-H10···O1	2.06	0.0197	0.0703	10.815	24.518	35.333	-3.650
		N3-H3···S2	2.59	0.0133	0.0361	5.618	12.495	18.113	-2.220
		C3-H6···S3	3.88	0.00673	0.0223	1.809	11.025	12.834	-0.759

[a] H···A represented the DFT calculated H···A distances

References

(1) Serge, A.; Joelle, R.; Fernand, S., Study of the antipyrine-sulfaguanidine system by x-ray diffraction and differential enthalpic analysis. *Ann. Pharm. Fr.* **1976,** 34, (3-4), 95-99.

(2) Alléaume, M.; Gulko, A.; Herbstein, F. H.; Kapon, M.; Marsh, R. E., Comparison of the dimensions and conformation of the sulfaguanidine moiety in sulfaguanidine monohydrate and trans-dichlorobis(sulfaguanidine)palladium(II). *Acta Cryst.* **1976,** B32, 669-682.

(3) Kálmán, A.; Czugler, M.; Argay, G., Conformational characteristics of anhydrous sulfaguanidine: computer retrieval and analysis of N-substituted arylsulfonamides. *Acta Cryst.* **1981,** B37, 868-877.
